# Harnessing genome-wide genetic diversity, population structure and linkage disequilibrium in Ethiopian durum wheat gene pool

**DOI:** 10.3389/fpls.2023.1192356

**Published:** 2023-07-20

**Authors:** Behailu Mulugeta, Rodomiro Ortiz, Mulatu Geleta, Teklehaimanot Hailesilassie, Cecilia Hammenhag, Faris Hailu, Kassahun Tesfaye

**Affiliations:** ^1^ Institute of Biotechnology, Addis Ababa University, Addis Ababa, Ethiopia; ^2^ Department of Plant Breeding, Swedish University of Agricultural Sciences, Alnarp, Sweden; ^3^ Sinana Agricultural Research Center, Oromia Agricultural Research Institute, Bale-Robe, Ethiopia; ^4^ Bio and Emerging Technology Institute, Addis Ababa, Ethiopia; ^5^ Department of Biology and Biotechnology, Wollo University, Dessie, Ethiopia

**Keywords:** domestication, durum wheat, landraces, nucleotide diversity, polymorphic information content, selection signature, single nucleotide polymorphisms

## Abstract

Yanyang Liu, Henan Academy of Agricultural Sciences (HNAAS), China; Landraces are an important genetic source for transferring valuable novel genes and alleles required to enhance genetic variation. Therefore, information on the gene pool’s genetic diversity and population structure is essential for the conservation and sustainable use of durum wheat genetic resources. Hence, the aim of this study was to assess genetic diversity, population structure, and linkage disequilibrium, as well as to identify regions with selection signature. Five hundred (500) individuals representing 46 landraces, along with 28 cultivars were evaluated using the Illumina Infinium 25K wheat SNP array, resulting in 8,178 SNPs for further analysis. Gene diversity (GD) and the polymorphic information content (PIC) ranged from 0.13–0.50 and 0.12–0.38, with mean GD and PIC values of 0.34 and 0.27, respectively. Linkage disequilibrium (LD) revealed 353,600 pairs of significant SNPs at a cut-off (*r^2^* > 0.20, *P* < 0.01), with an average *r^2^* of 0.21 for marker pairs. The nucleotide diversity (π) and Tajima’s D (TD) per chromosome for the populations ranged from 0.29–0.36 and 3.46–5.06, respectively, with genome level, mean π values of 0.33 and TD values of 4.43. Genomic scan using the *F_st_* outlier test revealed 85 loci under selection signatures, with 65 loci under balancing selection and 17 under directional selection. Putative candidate genes co-localized with regions exhibiting strong selection signatures were associated with grain yield, plant height, host plant resistance to pathogens, heading date, grain quality, and phenolic content. The Bayesian Model (STRUCTURE) and distance-based (principal coordinate analysis, PCoA, and unweighted pair group method with arithmetic mean, UPGMA) methods grouped the genotypes into five subpopulations, where landraces from geographically non-adjoining environments were clustered in the same cluster. This research provides further insights into population structure and genetic relationships in a diverse set of durum wheat germplasm, which could be further used in wheat breeding programs to address production challenges sustainably.

## 1 Introduction

Durum wheat (*Triticum durum* Desf.) is one of the most important crops cultivated worldwide, accounting for 10% (~17 million ha) of the total area used for growing wheat (Giraldo et al., 2016; Kabbaj et al., 2017; Zaïm et al., 2017; Sall et al., 2019). It is majorly produced in warm and semi-arid agro-ecozones (Kadkol and Sissons, 2016). The geographic regions where it is predominantly grown include the Mediterranean basin (providing 50% of world durum wheat production), North America, West Asia, and Eastern Africa (Kabbaj et al., 2017; Mérida-García et al., 2019). Among sub-Saharan countries, Ethiopia is the major durum wheat producer (Negisho et al., 2021; Mulugeta et al., 2022), contributing 18 to 20% of the country’s wheat production (Negisho et al., 2021).

Durum wheat was domesticated in the Fertile Crescent in the ninth millennium BC (Fayaz et al., 2019), and the Levantine region is considered a center of origin and diversity (Kabbaj et al., 2017). Some reports consider Ethiopia as the third country of domestication of durum wheat, which led to the development of *T. aethiopicum* and is regarded as the center of origin and diversity of tetraploid wheat, including *T. durum* (Mengistu et al., 2016; Kabbaj et al., 2017). Harlan (1969), Simmonds (1993), and Savage et al. (1994) also reported Ethiopia as a center of the astonishing diversity of tetraploid wheat species, which is evidenced by the presence of the crop wild relatives and diversified forms of these species in the country. Research has demonstrated the usefulness of Ethiopian durum wheat collection as a source of alleles for improving traits, including grain yield, nutritional quality, and host plant resistance to pathogens and drought tolerance (Mengistu et al., 2016; Kabbaj et al., 2017; Mengistu et al., 2018; Kidane et al., 2019; Alemu et al., 2020; Negisho et al., 2021; Mulugeta et al., 2023). For example, Mengistu et al. (2016) discovered new gene associated with days to booting, flowering and maturity. Kidane et al. (2019) found 177 unique protein‐coding genes in Ethiopian durum wheat utilizing a large nested association mapping population for breeding and quantitative trait locus mapping. Mulugeta et al. (2023) were also able to identify major novel loci associated to grain yield and related traits based on diverse sets of Ethiopian durum wheat landraces and cultivars. In spite of this, this valuable germplasm remains largely underutilized in breeding programs intended to improve these characteristics.

Analyses of the genetic diversity of crops is essential to determine the extent and pattern of diversity, domestication history, and the genetic relationship among different domesticated forms, such as landraces and cultivars (Soriano et al., 2016; Soriano et al., 2018; Rufo et al., 2019; Mazzucotelli et al., 2020). A comprehensive analysis of crop genetic diversity is necessary to enhance cultivar resilience to the changing climate. The genetic diversity of wheat under cultivation is declining sporadically due to its exposure to several bottlenecks during its domestication and post-Mendelian adoption of breeding, as well as due to the impacts of climate change and the growing human population (Louwaars, 2018; Pont et al., 2019; Kumar et al., 2020; Mazzucotelli et al., 2020; Sansaloni et al., 2020; Sthapit et al., 2020). To overcome these challenges, beneficial alleles can be transferred from crop wild relatives and landraces that are high in genetic diversity to improve the diversity of modern cultivars (Johansson et al., 2020; Kilian et al., 2020; Adhikari et al., 2022; Badaeva et al.). On the other hand, working with these genetic materials has challenges arising from the introduction of undesirable traits due to linkage drag, which needs careful selection to make them agronomically valuable for cultivar development programs (Mondal et al., 2016; Kilian et al., 2020; Sansaloni et al., 2020). Even if this limitation is challenging in crossbreeding, crop wild relatives and landraces remain the primary sources of novel beneficial alleles and diversity for future wheat improvement (Maccaferri et al., 2019; Sansaloni et al., 2020; Yadav et al., 2022). The determination of the extent and pattern of genetic diversity in durum wheat gene pool is therefore critical for future conservation and breeding efforts (Negisho et al., 2021).

Information on the population structure and linkage disequilibrium (LD) of the genetic materials of interest is also essential to understand the domestication and selection history, determine the genetic profiles of population subgroups (Jin et al., 2010; Tascioglu et al., 2016; Siol et al., 2017), and understand the evolutionary history of genomic regions (Maccaferri et al., 2010). These are crucial for providing a better understanding of genetic diversity in crop germplasm (Roncallo et al., 2021), and serve as the entry point for analyzing the genetic information of complex traits (Fiedler et al., 2017; Wang et al., 2019).

The extent and pattern of LD vary across populations, genetic regions, and proximity between pairs of loci (Fayaz et al., 2019). The LD between two loci decays progressively based on the degree of recombination rate and time passed across the number of generations (Fayaz et al., 2019; Maccaferri et al., 2019). The LD decay in plant species depends on the mutation rate, population size, the number of founding chromosomes in the population, and cycles of generation for which the population has existed (Devlin and Risch, 1995; Flint-Garcia et al., 2003; Roncallo et al., 2018). Research conducted so far to investigate the extent and pattern of population structure and LD in durum wheat germplasm has been very limited (Maccaferri et al., 2005; Fayaz et al., 2019; Liu et al., 2019; Alemu et al., 2020; Negisho et al., 2021; Roncallo et al., 2021). However, advances in genomic tools have played a pivotal role in estimating the extent and pattern of genetic variations, understanding the broader genetic implications of evolution, and executing hundreds of thousands of years’ effect of selection and breeding in durum wheat (Maccaferri et al., 2019; Sansaloni et al., 2020).

The investigation of the genetic diversity of Ethiopian durum wheat have been made previously based on phenotypic traits, which revealed high diversity and distinctness in its morphological characteristics (Eticha et al., 2006; Mengistu et al., 2015a; Dejene and Mario, 2016). More recently, the genetic diversity of Ethiopian durum wheat has been revealed using advanced genomic tools (Mengistu et al., 2016; Kabbaj et al., 2017; Mengistu et al., 2018; Asmamaw et al., 2019; Kidane et al., 2019; Kidane et al., 2019; Alemu et al., 2020). However, the germplasm used represents a tiny fraction of the existing durum wheat accessions in the Ethiopian Biodiversity Institute (EBI) gene bank (https://ebi.gov.et/resources/). In addition, only scanty research has previously analyzed the within-population genetic variation of Ethiopian durum wheat using recent genomic tools (Mengistu et al., 2016; Alemu et al., 2020; Negisho et al., 2021). The vast majority of *ex-situ* conserved Ethiopian durum wheat accessions have not been characterized using genome-wide DNA markers. Hence, molecular characterization of a large subset of the collections using such markers will facilitate the identification of exploitable and valuable genes and germplasm that can be utilized in crop improvement programs.

The present study aimed to evaluate the extent and amount of genetic diversity in diverse Ethiopian durum wheat landraces and cultivars. The study also aimed to describe genetic population structure and linkage disequilibrium in a set of durum wheat gene pools from Ethiopia, detect the admixture in a population, identify selection regions, and provide deeper insight into the level of genetic diversity and structure from different eco-geographic regions. This study highlights the ample amount of genetic diversity and untapped potential of Ethiopian durum wheat germplasm, which can be used to unravel novel genes for extending the gene pool and generating climate-resilient cultivars.

## 2 Materials and methods

### 2.1 Plant materials

**Figure 1:**
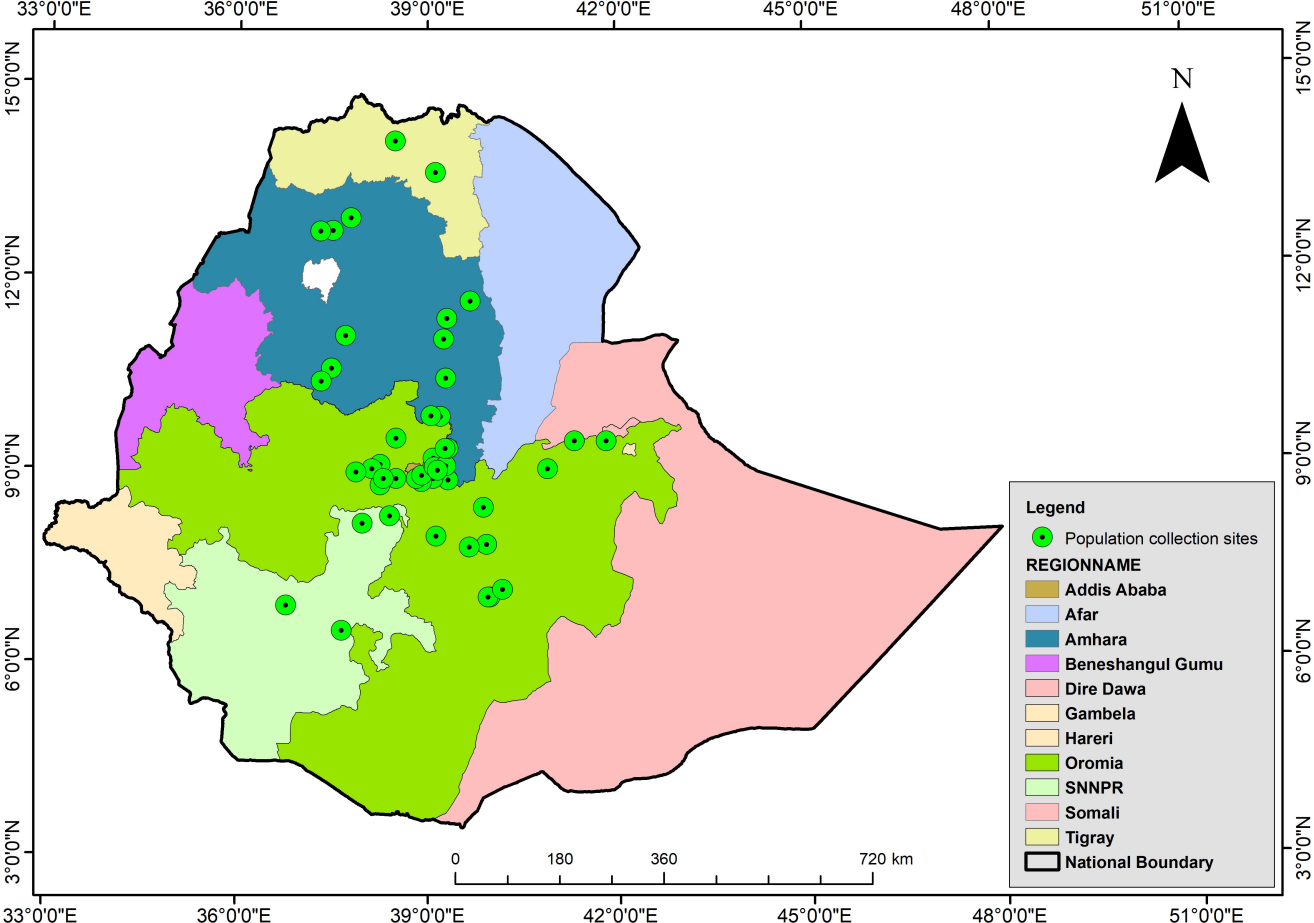
Map of Ethiopia indicating the geographical distribution of collection sites of 46 durum wheat landraces populations origin (shaded green) (NB: All boundaries are approximated and have nothing to do with political borders). The map was constructed using the ArcGIS software suite vs. 10.7.1.

This study examined 46 phenotypically diverse durum wheat landraces collected from various geographical regions of Ethiopia and 28 improved cultivars registered by the Ethiopian Ministry of Agriculture (MoA) after confirming their DUS (distinctness, uniformity, and stability) (**Figure 1**, **Supplementary Table 1**). Initially, the seeds of the landraces were obtained from EBI for phenotypic characterization. The landraces used in the present study were selected based on our previous phenotypic characterization (Mulugeta et al., 2022), which noticed a high within-landrace diversity in each landrace. Hence, phenotypically different landraces were selected to molecularly describe within-landrace variations. Each landrace was represented by 8 to 16 plants. Five hundred individuals representing the 46 landraces were individually sampled during field characterizations, along with 28 cultivars. Based on the information obtained in our previous study (Mulugeta et al., 2022), each landrace was considered as separate population. For simplicity, the landraces and modern cultivars were referred to as genotype. We represented all 28 cultivars as one separate population to see the level of genetic diversity existing in them.

### 2.2 Planting, leaf sample harvesting, and genomic DNA extraction

For each genotype (i.e., 528 samples representing 47 populations), five healthy seeds were randomly selected and planted in a square-shaped pot with a size of 10 cm × 10 cm × 11 cm in the greenhouse of the Swedish University of Agricultural Science (SLU) Alnarp, southern Sweden, for two weeks. Ten discs of young leaf samples pooled from five plants per genotype were harvested in 96-well deep well plates and freeze-dried using CoolSafe ScanVAC Freeze Dryer following the instruction of TraitGenetics. The freeze-dried leaf samples were sent to Trait Genetics (Gmbh, Gatersleben, Germany) for genomic DNA extraction and subsequent genotyping. The genomic DNA was extracted using a standard cetyltrimethylammonium bromide (CTAB) method from the leaf samples using TraitGenetics’ lab protocol.

### 2.3 SNP selection, genotyping, and filtering of SNP markers

The samples were genotyped using a high-density Illumina Infinium 25k wheat single nucleotide polymorphism (SNP) array by TraitGenetics Gmbh (Gatersleben, Germany). This SNP array contains most SNPs from the earlier 90k Infinium array, 35K Wheat Breeders array, 135K Axiom wheat array, and SNPs within genes associated with specific importance in durum wheat breeding. SNPs accurately matching the A and B genomes were selected based on a cluster file of hexaploid wheat and the details of these SNPs can be found at https://sgs-institut-fresenius.de/en/gesundheit-und-ernaehrung/traitgenetics/genotyping. The SNP loci were filtered by removing those with a missing value above 5% and a minor allele frequency (MAF) below 5% using TASSEL v 5.2.67 software (Bradbury et al., 2007). These filtering steps resulted in 8,178 SNPs for further genetic information analysis.

### 2.4 Data analysis

#### 2.4.1 Patterns of genomic nucleotide variations

The nucleotide diversity (π) (Nei, 1987) and Tajima’s D (Tajima, 1989) of each population were analyzed using the PopGenome package (Pfeifer et al., 2014) in the R program (R Development Core Team, 2021) to uncover genome-wide genetic variation. The sliding window approach with a window size of 1,000 kbp and a jump size of 100 kbp was applied as previously described (Liu et al., 2019). The site frequency spectrum of each population was analyzed using the software DnaSP version 6 (Rozas et al., 2017). The number of alleles (Na), the mean number of effective alleles (Ne), Shannon’s information index (*I*) and Hardy-Weinberg equilibrium (HWE) test were performed using the GenAIEx v.6.5 software (Peakall and Smouse, 2012). The polymorphism information content (PIC) (Serrote et al., 2020) and gene diversity were computed using Power marker v3.25 (Liu and Muse, 2005). The observed heterozygosity (*Ho*), expected heterozygosity (Nei, 1973), and the percentage of polymorphic loci (PPL) were analyzed using Arlequin v.3.5.2.2 (Excoffier and Lischer, 2010).

Loci under selection from genome scans were analyzed assuming a null distribution under the hierarchical island model with 100,000 simulations and 100 numbers of demes simulated per population as described in Excoffier and Lischer (2010) using Arlequin v.3.5.2.2 (Excoffier and Lischer, 2010). Comparative analyses with previously published reports using different *Triticum* databases including GrainGene, T3/wheat, and Wheat URGI were used to determine the potential genes associated with loci under selections that are controlling important traits (Alaux et al., 2018). To identify genes related to selection signatures, lists of identified putative candidate genes and their functions were downloaded from the NCBI database (https://ftp.ncbi.nlm.nih.gov/genomes/all/GCA/900/231/445/GCA_900231445.1_Svevo.v1/).The nucleotide position extending from 1–8.56 Mbp up and downstream from the SNP position was used for searching the potential candidate genes, as previously reported for wheat ((Breseghello and Sorrells, 2006). The genes associated with the regions under selection signatures were obtained from the durum wheat (*Triticum turgidum* (Svevo.v1)) reference genome (Maccaferri et al., 2019).

#### 2.4.2 Linkage disequilibrium (LD) analysis

Knowing LD among pairs of multiple SNP markers provides valuable information on the correlation structure of different loci based on their allelic variation (Siol et al., 2017). The pairwise LD (measured as *r^2^*) for SNP pairs was calculated as described in Weir (1997) using TASSEL version 5.2.8 (Bradbury et al., 2007) based on the LD window size of 50 bp. The decay rate was estimated for significant SNP marker pairs (*r^2^ = *0.20, *p<*0.01) for A and B genomes separately as well as for the whole genome. The association of genome-wide LD decay and the physical distance was plotted by fitting a locally weighted linear regression (loess) line using the R function ‘loess`(R Development Core Team, 2021). The physical distance at which the *r^2^* value dropped to half its average maximum value was considered the LD decay rate (Huang et al., 2010).

#### 2.4.3 Genetic population structure analysis

Principal Coordinate Analysis (PCoA) based on Nei’s standard genetic distance was also performed to investigate further the association between the populations using GenAIEx v.6.5 (Peakall and Smouse, 2012). A Bayesian Model-based clustering algorithm implemented in the software STRUCTURE version 2.3.4 (Pritchard et al., 2000) was utilized to infer the population genetic structure. An ADMIXTURE model and correlated allele frequencies were assumed to assess the ancestry fractions of each subgroup attributed to each landrace. The burn-in period and Markov Chain Monte Carlo (MCMC) iterations for subgroups (K) ranging from K1 to K10 independent runs were adjusted to 50,000 and 100,000, respectively. The program STRUCTURE Harvester (Earl and von Holdt, 2012) was used to visualize the results. The best K representing the germplasm analyzed was determined using the delta K (ΔK) method as described in Evanno et al. (2005), and the optimum K bar plot was drawn using the CLUMPAK online server (Kopelman et al., 2015). Genotypes with an arbitrary value of *Q > 75%* of their genome were regarded as pure genotypes, while those with membership probabilities *Q < 75%* for each genotype were considered admixture (Carović-Stanko et al., 2017). Nei’s standard genetic distance (Nei, 1973) based unweighted pair group method with arithmetic mean (UPGMA) cluster analysis was performed using Power Marker v.3.25 (Liu and Muse, 2005) to determine the relationship between the populations further. Software MEGA version x (Kumar et al., 2018) was used to visualize the UPGMA tree. Analysis of molecular variance (AMOVA) was performed to partition the total genetic variation into variation within individuals, among individuals within populations, and among populations and groups (Weir and Cockerham, 1984; Peakall and Huff, 1995) using the software Arlequin v.3.5.2.2 (Excoffier and Lischer, 2010). Arlequin was also used to estimate pairwise genetic variation within populations and differentiation among populations. The joint population differentiation (*F_ST_*) distribution and heterozygosity were analyzed as described in Excoffier and Lischer (2010).

## 3 Results

### 3.1 SNP markers’ quality, distribution, density, and levels of polymorphism

**Figure 2:**
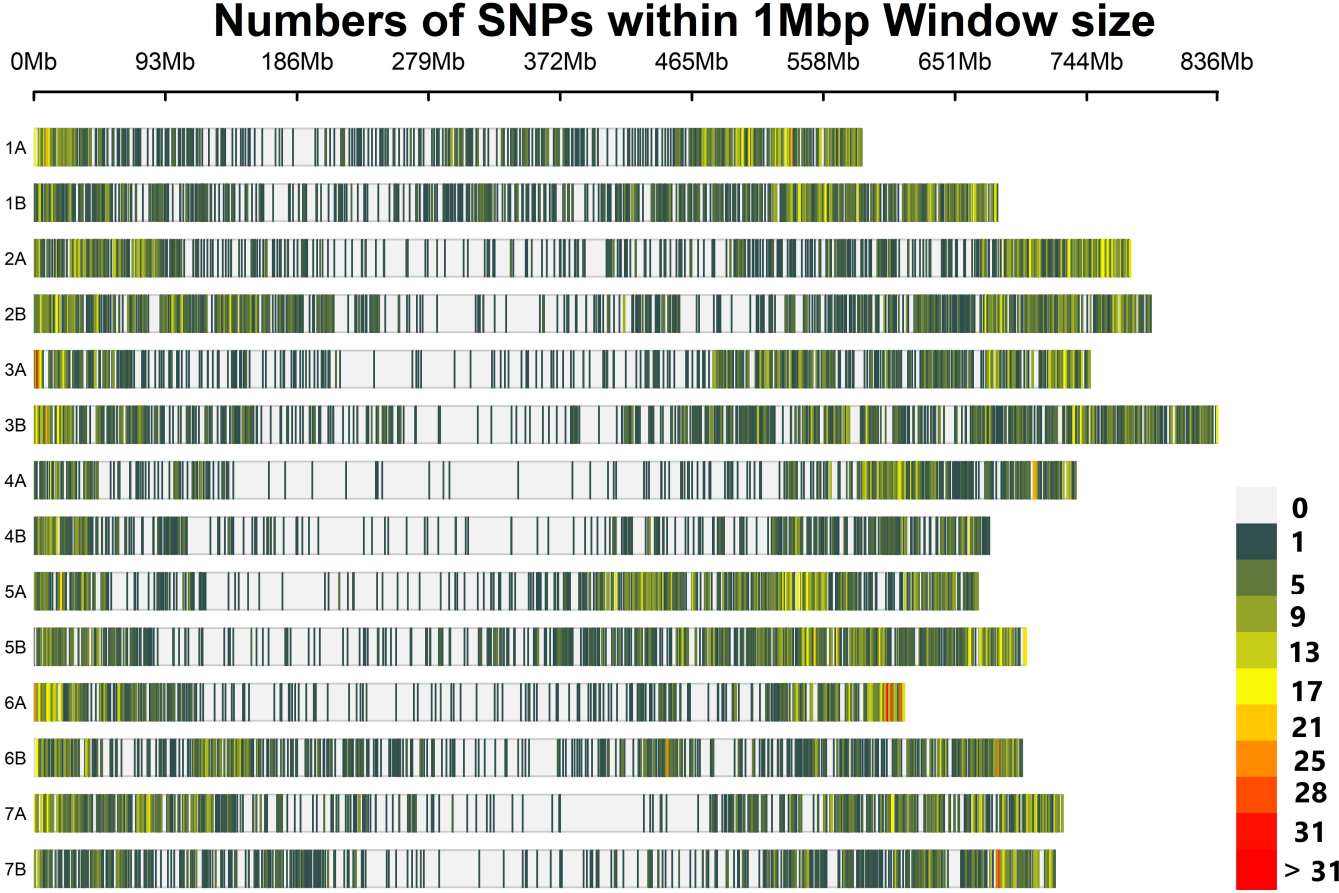
The density and distribution of the SNP markers used for genotyping in the present study on each durum wheat chromosome. The heatmap scales show the density of the markers per Mbp.

From a total of 24 145 SNP markers, after removing SNP markers with a missing value above 5% and MAF below 5%, 8,178 polymorphic and high-quality SNP loci distributed across all 14 durum wheat chromosomes were selected for further genetic analysis. Of these 8,178 SNP markers, 3,471 (42.4%) and 3658 (44.7%) have known map positions on the A and B genomes, respectively (**Table 1**). The map positions of 1049 (12.83%) SNPs on the durum wheat genome have not been precisely determined. Chromosomes 5B and 4B contained the highest and lowest number of SNPs per chromosome, with 659 SNPs and 290 SNPs, respectively (**Figure 2**; **Table 1** **)**. The average marker density was 0.72, 0.73, and 0.72 markers per Mbp for the A and B genomes and the whole genome, respectively. In total, the distribution of these SNP markers covered 9.85 Gbp regions of the durum wheat genome, with chromosomes 1A and 2B having the least (582.20 Mbp) and largest (788.36 Mbp) regions covered (**Figure 2**; **Table 1**).

**Table 1:** The distribution of the 8,178 SNP markers across the durum wheat genome.

Chr^z^	NLChr	GCR (Mbp)	SCGR (Mbp)	GD	PIC	Chr^z^	NLChr	GCR (Mbp)	SCGR (Mbp)	GD	PIC
1A	576	1.10-583.30	582.20	0.34	0.28	1B	602	3.29-681.10	677.81	0.36	0.29
2A	484	0.29-774.80	774.50	0.32	0.26	2B	595	1.06-789.42	788.36	0.32	0.26
3A	414	0.30-744.80	744.50	0.36	0.29	3B	588	0.30-830.24	829.94	0.29	0.24
4A	344	0.69-736.50	735.81	0.34	0.27	4B	290	0.04-673.81	673.77	0.34	0.27
5A	535	0.27-667.30	667.03	0.32	0.26	5B	659	2.59-701.17	698.58	0.34	0.27
6A	559	0.59-613.94	613.35	0.34	0.28	6B	495	2.05-695.38	693.33	0.34	0.27
7A	559	0.17-727.02	726.85	0.34	0.28	7B	429	0.43-721.08	720.65	0.33	0.27
A^a^	3471	na	4,844.24	0.34	0.27	B^b^	3,658	na	5,008.44	0.33	0.27
Unmapped	1069										

Chr^z^, Chromosome; A^a^, A genome; B^b^, B genome; bp, Base pair; Mbp, Mega base pair; NLChr, Number of loci per chromosome; GCR, Genome coverage range; SCGR, Size of covered genomic region; GD, Gene diversity, PIC, Polymorphism information content, na, Not applicable.

The minor allele frequency (MAF) of the 8,178 SNP loci ranged from 0.07 to 0.5 with a mean of 0.24. The levels of polymorphism measured in terms of gene diversity (GD) ranged from 0.13 (at 505 loci) to 0.50 (at 2345 loci) with a mean gene diversity of 0.34. At a chromosome level, GD ranged from 0.32 to 0.36 with a mean value of 0.33 across the genome (**Table 1**). The PIC, an indicator of the informativeness of markers, ranged from 0.12 (for 208 loci) to 0.38 (for 456 loci), with a mean PIC value of 0.27. Moreover, at the chromosome level, the PIC varied from 0.24 on chromosome 3B to 0.29 on chromosomes 1B and 3A, respectively (**Table 1**). The expected heterozygosity (*He*) value across all loci ranged from 0.02 to 0.18.

### 3.2 Magnitude and pattern of allelic diversity in the populations

Several molecular diversity indices were determined to evaluate the magnitude and pattern of within-landraces genetic variation of the 46 landraces. The observed number of alleles (Na) and the effective number of alleles (Ne) per locus of the landraces varied from 1.00 (EH2) to 1.75 (WSH8) and from 1.00 (EH1) to 1.31 (WSH8), respectively. The mean Na and Ne values were 1.30 and 1.10, respectively. The highest percentage of polymorphic loci (%P, 85.65%) was found in cultivars population, followed by landrace WSH7 (%P, 74.5%), WSH3 (71.51%) and WSH8 (64.67%) (**Supplementary Table 2**). In contrast, landraces EH2, NSH4, NSH8, and WSH2 had no or almost no polymorphic loci, with %P of 0.00, 0.01%, 0.02%, and 0.02%, respectively. The mean %P across all landraces was 31.5%. The Shannon information index (*I*) for the landraces ranged from 0 (for EH2) to 0.33 (for WSH8), with a mean of 0.11. The observed heterozygosity (*Ho*) values were from 0 for landrace EH2 to 0.07 for landrace BL1, with a mean value of 0.011. The expected heterozygosity (*He*) of the landraces varied from 0 for EH2 to 0.21for NSH8, with a mean value of 0.07. The gene diversity for the landraces ranged from 0 for nine of the 46 landraces to 0.22 for WSH8, with a mean value of 0.07 (**Supplementary Table 2**).

There was a wide range of variation of molecular diversity of the SNP loci. The Shannon Information Index (*I*) ranged from 0.02 to 0.26, with a mean value of 0.12 (**Supplementary Table 3**). The Ho across the loci varied from 0.00 to 0.28, with a mean of 0.01. He and uHe across the loci ranged from 0.01 to 0.18, with a mean value of 0.07 for both indices. The gain (increased *He*) and loss of heterozygosity (increased *Ho*) were recorded for 99.9% and 0.1% of the loci, respectively. The fixation indices showed wide variation between the SNP loci. The fixation indices’ minimum, maximum, and mean were -0.66, 1.00, and 0.84 for *F_IS_*, 0.04, 1.00, and 0.96 for *F_IT_*, and 0.39, 0.96, and 0.76 for *F_ST_*, respectively (**Supplementary Table 3**).

The Hardy Weinberg Equilibrium (HWE) test was carried out for all SNP loci for each landrace (population) as well as for all landraces. Almost all of the SNP loci (99.9%) significantly deviated from HWE across landraces (*p<0.01*). Almost all their loci (99%) significantly deviated (99.9%), thus showing heterozygote deficiency, which is in agreement with the inbreeding reproductive system of durum wheat. Only 0.1% (8 loci) had excess heterozygosity (**Supplementary Table 3**). Based on the HWE proportion, we categorized the landraces into two subgroups. The first group contains 26 landraces, whose genotypic proportions at most of the SNP loci significantly deviated from the HWE. The second group comprised 18 landraces, and more than half of the SNP markers hold the assumptions of HWE. For example, landraces NSH6, WGM2, NO, NSH2, AR1, and BL1 held the assumptions of HWE for 5074, 2217, 2165, 1951, 1760, and 1299 SNPs markers from respective polymorphic loci within each of these landraces, respectively. For landrace NSH6, 98.8% of loci hold the assumptions of HWE. Landraces WSH2, WSH5, WSH6, and ESH3 revealed only 2 to 3 polymorphic loci out of 8,178 SNP markers. Interestingly, these loci exhibited excess heterozygosity with a significant deviation from the HWE assumption (*p<0.05*).

### 3.3 Pattern and extent of linkage disequilibrium (LD)

**Figure 3:**
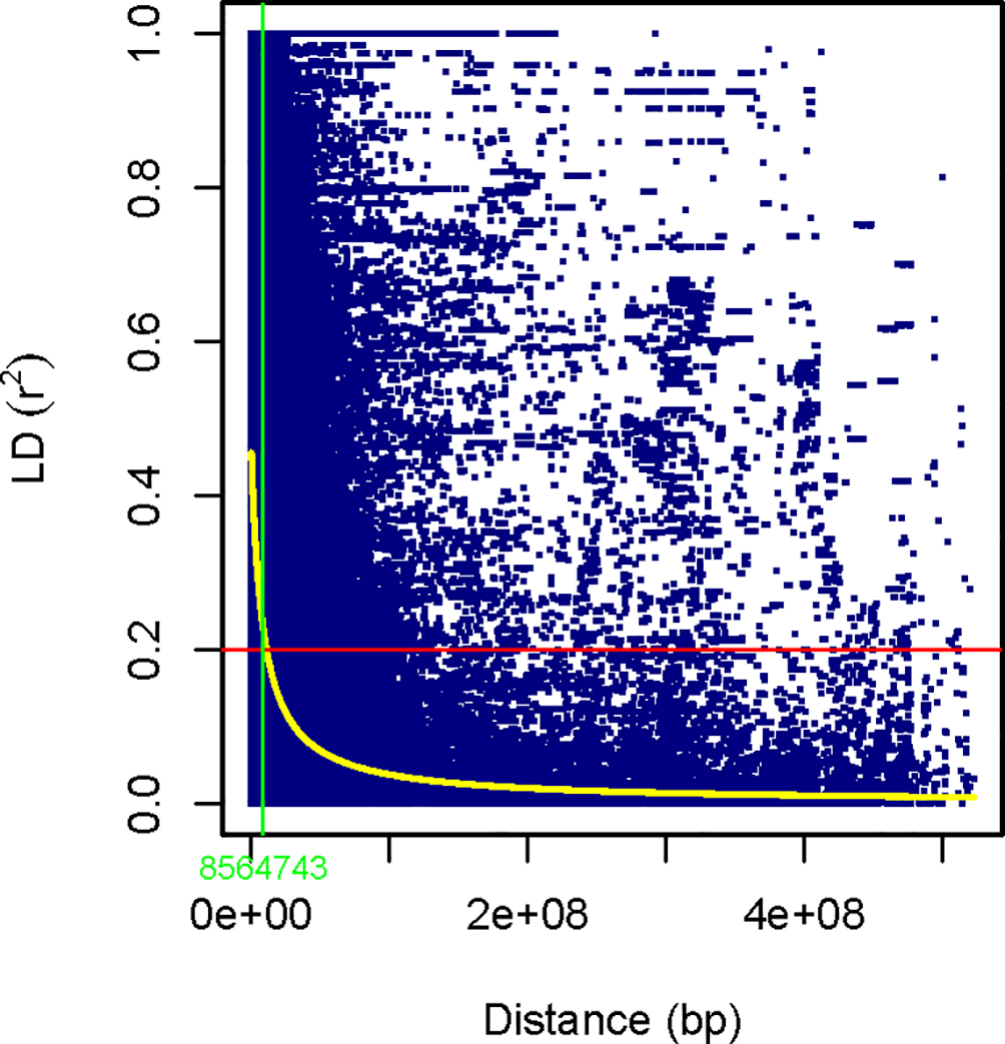
Scatter plot of genome-wide LD decay against total physical distance (bp) based on the *r^2^* values of the marker pairs. The horizontal red line represents the half decay r2 value of the genome (*r^2^ = *0.2). The yellow curve line is the smoothing spline regression model fitted to LD decay. The vertical light green line in bp (8,564,743bp) indicates the intersection between the half decay and the LD decay curve.

The extent of LD (*r^2^*), measured as the squared correlation of alleles at two loci, was estimated based on 7,129 SNPs in durum wheat genotypes since 1,049 SNPs do not have known positions on the durum wheat chromosome. Considering the whole genome, 353,600 pairs of SNPs were in LD and 107,471 (30.4%) were significant marker pairs at *p*<0.01 (*r^2^* ≥ 0.2; **Table 2**). The number of significant marker pairs ranged from 5,236 (18.7%) on chromosome 7A to 11,554 (39.3%) on chromosome 3B. The average *r^2^* value for marker pairs in LD on each chromosome varied from 0.14 (on chromosome 7A) to 0.26 on chromosome 3B (**Table 2**), with a mean *r^2^* value of 0.21 for the whole genome. As the physical distance between marker pairs increased on each chromosome, the mean *r^2^* values of the SNP pairs rapidly declined. The LD decay (at cut-off *r^2 ^= *0.2) of pairs of markers happened within the range of 3.65 Mbp on chromosome 4A to 22.90 Mbp on chromosome 3B, with a mean of 8.56 Mbp across the genome (**Figure 3**).

**Table 2:** Chromosome (Chr), number of SNP markers per Chr (NSMpC), the total number of LD pairs (TNLP), mean r^2^ value of all pairs (MRAP), numbers of significant SNP pairs (NSSP), mean r^2^ for all significant pairs (MASP, r^2^ > 0.20 at *P < 0.01*), percent of significant pairs (%SP), numbers of pairs in complete LD (NPCL), LD decay in Mbp (LDD Mb), Nucleotide diversity (ND (π)) and Tajima’s D (TD) for all chromosomes, A and B genomes, and for the whole genome.

Chr	NSMpC	TNLP	MRAP	NSSP	MASP, r^2^ > 0.20 at *P < 0.01*	%SP	NPCL	LDD (Mbp)	ND (π)	TD
1A	576	28800	0.2092	8470	0.5899	29.41	1270	5.81	0.342	4.603
1B	602	30100	0.2035	9467	0.5276	31.45	709	7.90	0.363	5.063
2A	484	24200	0.1761	6137	0.5596	25.36	608	4.88	0.323	4.189
2B	595	29750	0.1874	8638	0.5258	29.04	706	9.40	0.324	4.210
3A	414	20700	0.1796	5413	0.5347	26.15	580	6.92	0.357	4.925
3B	588	29400	0.2573	11554	0.5713	39.30	786	22.90	0.290	3.464
4A	344	17200	0.1435	3793	0.4573	22.05	241	3.65	0.338	4.482
4B	290	14500	0.2032	4501	0.5336	31.04	331	15.30	0.334	4.419
5A	535	26750	0.2411	9785	0.5616	36.58	1001	14.26	0.319	4.096
5B	659	32950	0.2125	11266	0.5121	34.19	956	8.76	0.341	4.585
6A	559	25100	0.2625	9395	0.6123	37.43	1715	9.10	0.345	4.665
6B	495	24750	0.2066	8055	0.5168	32.55	703	11.78	0.341	4.578
7A	559	27950	0.1392	5236	0.5240	18.73	519	4.06	0.336	4.483
7B	429	21450	0.1708	5762	0.4912	26.86	369	7.87	0.329	4.314
Genome A	3471	171700	0.1957	48,228	0.5609	28.09	5,929	6.66	0.337	4.492
Genome B	3,658	182,900	0.2077	59,243	0.5283	32.39	4,559	10.86	0.332	4.376
Whole genome	7,129	353,600	0.2019	107,471	0.5429	30.39	10,489	8.56	0.334	4.434

### 3.4 Genomic pattern of nucleotide variation

**Figure 4:**
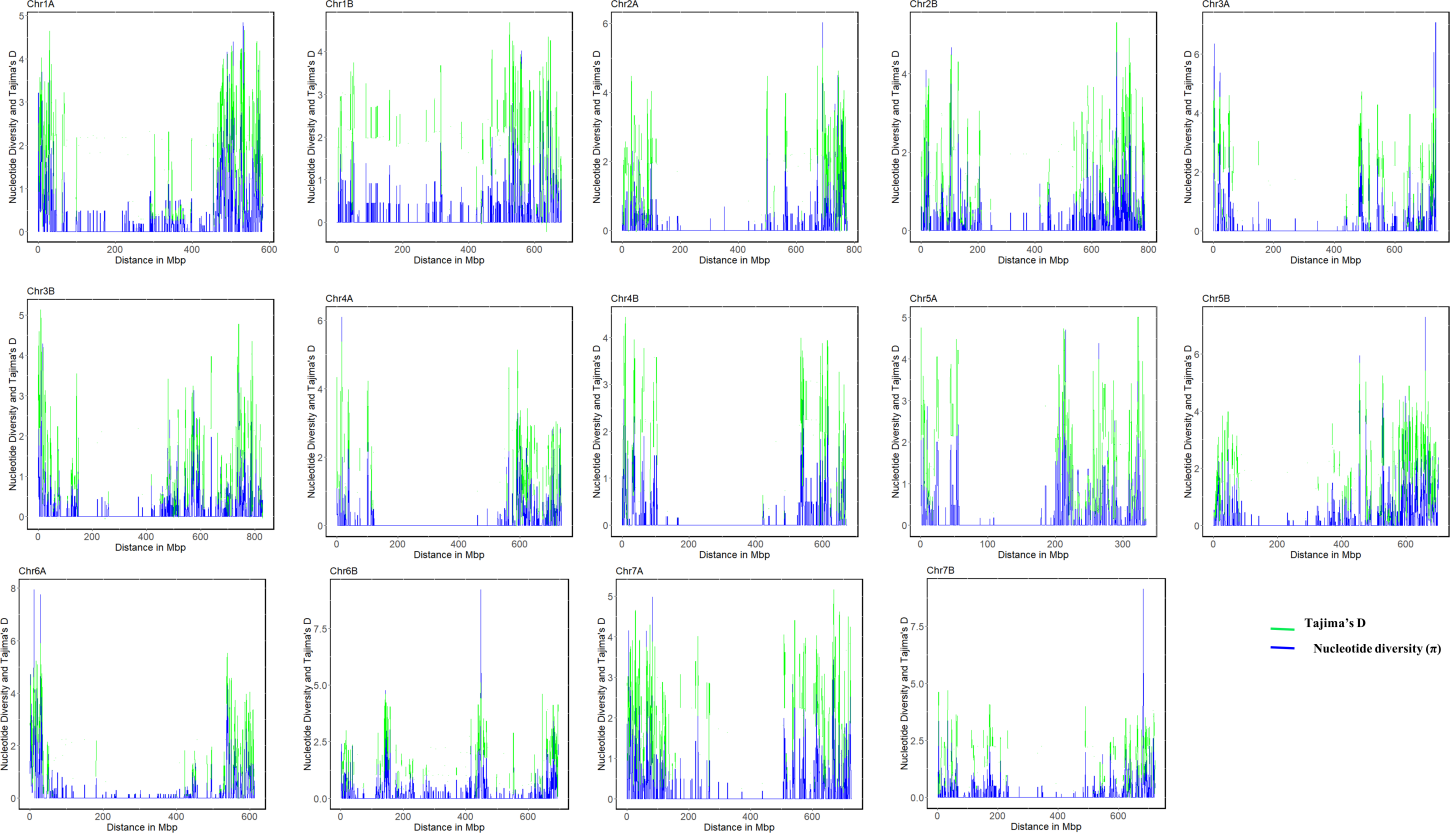
Genome-wide pattern of nucleotide diversity (ND) and Tajima`s diversity (TD) of all population of 46 durum wheat landraces based on the sliding window approach with a window size of 1000 kbp and jump size of 100 kbp.

Genome-wide variation and selection signature in Ethiopian durum wheat were examined with nucleotide diversity and Tajima’s D. The mean nucleotide diversity (π) per chromosome varied from 0.29 on chromosome 3B to 0.36 on chromosome 1B, with an average π value of 0.33 (**Table 2**). Most of each chromosome’s pericentromeric regions exhibited a significant loss of variation in nucleotide diversity except for chromosomes 1A, 1B, 6A, and 6B, which exhibited wide variation across their chromosomes. In contrast, the distal regions of each chromosome had high nucleotide diversity (**Figure 4**), suggesting the presence of balancing selection in these regions. The A genome exhibited higher mean nucleotide diversity than the B genome (**Table 2**). At the population level, π value varied from 1 × 10^-5^ (for population EH2) to 22 × 10^-2^ (for populations AR4 and cultivars), with the overall population π, a mean value of 34 × 10^-2^, which indicated a wide genetic variation among the populations (**Supplementary Table 2**).

The highest (5.06) and lowest (3.5) mean Tajima’s D were recorded for chromosomes 1B and 3B, respectively, with Tajima’s D mean of 4.4 across the whole genome (**Table 2**). The pattern and extent of variation in Tajima’s D across each chromosome nearly match the pattern of nucleotide diversity (**Figure 4**). Higher diversity in Tajima’s D was observed in the distal regions than in the proximal regions of all 14 chromosomes (**Figure 4**), which revealed reduced levels of genetic diversity around the proximal (pericentromeric) regions of the chromosomes. Tajima’s D values for the landraces ranged from -2.86 (NSH2 and NO), indicating population expansion, to 2.38 (NSH6). The mean value of Tajima’s D across all landraces was 4.50 (**Supplementary Table 2**). Using nucleotide diversity (π) and Tajima’s D, these results exhibited strong signatures of genetic divergence associated with domestication and breeding on chromosomes 1A, 1B, 6A, and 6B than on other chromosomes of the A and B genomes.

The number of segregating variants at different levels of allele frequency in a population was estimated based on the site frequency spectrum (SFS) to infer the joint distribution of observed and expected allelic frequencies. The SFS analysis revealed considerable variation in the minor allele frequency (MAF) distribution of all SNP loci across the landraces (**Supplementary Figures 1A–C**). A coalescent analysis approach exhibited a disparity of joint distributions of expected and observed allelic frequency across most individuals in the population except for P4 (WSH3), P19 (JM), P44 (WSH8), and P47 (cultivars), which exhibited moderate matching of both observed and expected allelic distributions (**Supplementary Figure 1A–C**). The populations’ haplotype diversity also ranged from 0.10 for NSH4 to 1.00 for WSH3, WSH8, TG1, and cultivars, being the population-wise haplotype diversity of 1.00.

### 3.5 Selection signatures and identified putative regions

**Figure 5:**
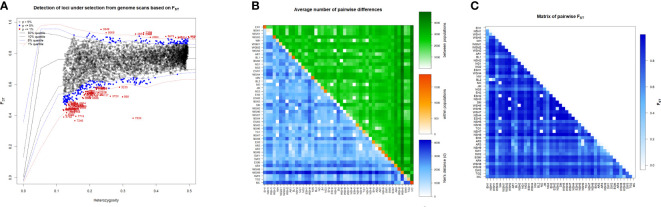
Graphical depictions revealed based on 8178 SNPs markers for **(A)**. Detected loci under selection from genome scan based on FST, SNPs colored with red is significant loci under selection at *p<0.01* and blue color indicate significant loci under selection at *p<0.05*, **(B)** Heatmap presenting average number of pairwise differences of the 47 durum wheat populations, estimated using a number of different alleles as a distance method: average number of pairwise differences between the landraces (above diagonal), average number of pairwise differences within the corresponding landrace (diagonal); and corrected average pairwise difference (below diagonal), and **(C)** Heatmap signifying pairwise genetic differentiation (FST) among the 47-durum wheat population calculated using the number of different alleles as a distance method. The differentiation between each pair of landraces was significant (*p* < 0.05) except in the case of pairs marked with a purple asterisk.

Among the 8,178 informative SNPs used to scan for loci under selection, 85 loci at 1% quantiles (significant at *p<*0.01) were regarded as loci under selection, covering all 14 chromosomes of the durum wheat genome (**Supplementary Table 4**; **Figure 5A**). Of these, 65 loci were outliers with lower *F_st_* values ranging from 0.36 to 0.58 and were regarded as candidate loci putatively subjected to under-balancing selection. In contrast, 16 loci have high *F_st_* values varying from 0.89 to 0.95 and were putative candidate loci under local directional selection. The putative loci under balancing selection span across all 14 chromosomes, whereas those under directional selection are located on chromosomes 2A, 3A, 5B, 6B, and 7B. Higher numbers of loci under selection were recorded for B genome chromosomes than for A genome chromosomes. Candidate genes located near the selection signatures were identified by searching the genomic regions of loci under selection against the Svevo durum wheat reference genome (Maccaferri et al., 2019) using an interval of ± 8.6 Mbp, which is the average LD decay of the whole genome.

Some of the identified candidate genes that are co-localized with the loci under selection are *TRITD2Bv1G218450* (heavy metal-associated protein), *TRITD2Bv1G029100* (heat shock transcription factor), *TRITD3Av1G181000* (E3 ubiquitin-protein ligase SDIR1 G), *TRITD5Bv1G162250* (sugar transporter ERD6), *TRITD5Bv1G162180* (disease resistance protein (TIR-NBS-LRR class) family), *TRITD3Bv1G028390* (30S ribosomal protein S7), *TRITD5Bv1G155770* (60S ribosomal protein L32), *TRITD5Bv1G198940* (photosystem II protein), *TRITD5Bv1G236030* (high affinity nitrate transporter), *TRITD7Bv1G197270* (MADS-box transcription factor G), *TRITD6Bv1G138770* (MYB transcription factor 1), and *TRITD7Bv1G165520* (zinc finger CCCH zinc-finger proteins) (**Supplementary Table 5**).

### 3.6 Population structure and genetic relationship between populations

**Figure 6:**
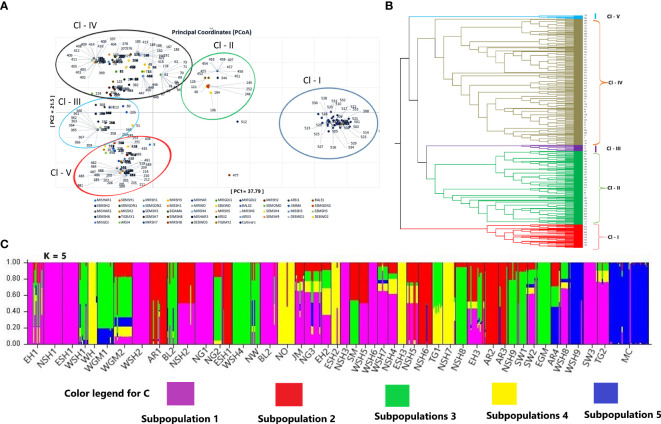
Principal coordinate analysis (PCoA) generated based on Nei’s unbiased genetic distance, representing the relationship between the genotypes **(B)** Unweighted pair group method with arithmetic mean (UPGMA) tree showing the genetic relationship, and **(C)** the population genetic structure of the genotypes at K = 5. The five colors represent the five clusters, and the proportion of each color in each landrace represents the average proportion of the alleles that placed each landrace under the five clusters.

Principal coordinate analysis (PCoA), UPGMA, and model-based Bayesian Inference were used to determine the population structure and genetic relationship between the landraces. The first three principal components (PCs) of PCoA explained 67.3% of the total variation, with the first two PCs (PCo1 = 37.79%, PCo2 = 21.50%) capturing 59.29% of the total variation. The PCoA grouped the landraces into five major clusters (**Figure 6A**). There was no correlation between the geographical origin of the landrace and their clustering within the first four clusters determined by the PCoA. The fifth cluster contained almost all modern cultivars.

The UPGMA tree, following the average linkage algorithm, agreed with the grouping pattern generated through PCoA analysis and grouped the genotypes into five distinct clusters (**Figure 6B**). Cluster 1 includes all modern cultivars (28) and 25 genotypes of populations from Arsi, East Shewa, and West Shewa. Cluster 2 was the second largest cluster containing 170 genotypes (31.91%) of populations from Arsi, Bale, East Shewa, East Gojem, Sidama, North Gonder, East Hararge, West Shewa, North Shewa, North Omo, North, West, and South Wollo. Cluster 3 was the only cluster comprising 14 genotypes from a single population (WGM2). Cluster 4 was the largest and most diverse, comprising 289 genotypes (54.2% of all genotypes) of populations from Arsi, Bale, East Shewa, East Gonder, Jimma, North Gonder, West Hararge, West Shewa, North Shewa, North Omo, and South Wollo. Genotypes in clusters 2 and 4 were highly diverse, and their grouping did not follow the geographical regions of origin of their landraces. Cluster 5 comprised the least number of genotypes (eight) of populations from Bale, West Shewa, North Omo, and East Hararge (**Supplementary Table 1**).

Bayesian Model-based population structure analysis revealed the highest ΔK value at K = 2, followed by K = 5, suggesting the optimal biological Inference into two and five subgroups, respectively. The number of clusters of five (K = 5) (**Figure 6C**) was then considered optimal since it agreed with the number of clusters obtained through PCoA and cluster analyses. For K = 5, Cluster 1 (Cl-I) comprised 28 cultivars and 33 genotypes from Arsi, West Shewa, North Wollo, and North Gonder populations. Cluster 2 (Cl-II) included 137 (25.65% of the genotypes) populations from Arsi, Bale, East Hararge, East Gojam, North Gonder, North Wollo, West Gojam, West Shewa, West Wollo, Sidama, Tigray, and South Wollo. Cluster 3 (Cl-III) comprised 67 (12.54% of the genotypes) populations from Arsi, East Shewa, North Shewa, West Shewa, West Hararge, and North Omo. Cluster 4 (Cl-IV) was the largest, comprising 218 genotypes (40.82%) of populations from Arsi, Bale, East Gojam, East Hararge, Jimma, North Gonder, North Shewa, South Wollo, Tigray, West Hararge, and West Shewa. Cluster 5 (Cl-V) comprised 51 genotypes (11.42% of the genotypes) of populations West Hararge, West Gojem, North Omo, North Shewa, and Tigray. Compared to PCoA and UPGMA, this Bayesian-based population structure analysis grouped the genotypes slightly better regarding their geographical regions of origin. The analysis to determine whether a genotype is pure or admixed based on the Q value score (*Q < 0.75* = admixture, and *Q > 0.75* = pure genotypes) revealed that 177 genotypes (149 from landrace landraces and 28 cultivars) were admixed (**Figure 6C**).

The net nucleotide (allelic) divergence among the subgroups inferred by STRUCTURE showed that the highest allelic divergence (0.47) was observed between clusters 1 and 3, whereas the lowest (0.24) was observed among clusters 4 and 5. The average genetic distance between genotypes in the same clusters ranged from 0.01 (Cluster 5) to 0.26 (Cluster 2). The mean expected heterozygosity between genotypes in the same clusters for cluster 1, cluster 3, and cluster 4 was 0.19, 0.10, and 0.13, respectively. The mean *F_st_* values of the subgroups varied from 0.53 for cluster 2 to 0.99 for cluster 5. The mean *F_st_* values for clusters 1, 3, and 4 were 0.68, 0.83, and 0.79, respectively.

### 3.7 Genetic differentiation of the hierarchical populations and gene flow

Analysis of molecular variance (AMOVA) was used to infer hierarchical genetic differentiation and estimate genetic variation within individuals, within populations, and among populations. The analysis revealed highly significant genetic differentiation among populations (*F_st_* = 0.77, *p*<0.001), which accounted for 76.68% of the entire genetic variation. Genetic variation among individuals within populations accounted for 20.18% of total genetic variation. The genetic differentiation between groups of populations that were grouped according to their Regional States of origin accounted for 1.18% of the total genetic variation (*F_CT_* = 0.012, *p*< 0.341), 76.66% among populations within the Regional States (*F_SC_* = 0.77, *p*<0.001) and 22.17% among individuals within populations (*F_st_* = 0.78, *p*<0.001), indicating high genetic variation among populations and individuals within the Regional States and absence of genetic differentiation among Regional State-based groups. AMOVA carried out by grouping the populations according to their geographical locations of origin revealed that 75.42% of the entire variation exists among populations within geographical regions of origin (*F_SC_* = 0.77, *p<*0.001*)*, 23.04% among individuals within populations (*F_st_*: 0.76, *p<*0.001) and 1.54% among geographical regions of origin (*F_CT_* = 0.02, *p=0.312*). According to the AMOVA for the five STRUCTURE-based subpopulations, 44.50% of the total genetic variation was found between the five subpopulations and 52.62% among individuals within the subpopulations (**Table 3**).

**Table 3:** Analysis of molecular variance for durum wheat populations at different hierarchical levels without grouping the populations and by grouping the populations according to their Regional States, geographical locations, and Bayesian Model-based (STRUCTURE) clusters.

Grouping Method	Source of variation	DF†	SS	VC	PV	FI	ST
Original populations (Landraces)	Among populations	46	1115752.76	1055.88 Va	76.78	FIS=0.86	Va and FIS (*P < 0.001*)
	Among individuals within populations	481	285861.11	274.99 Vb	20.00	FST=0.77	Vb and FST (*P < 0.001*)
	Within individuals	528	23396	44.31 Vc	3.22	FIT=0.97	Vc and FIS (*P < 0.001*)
	Total	1055	1462454.66	1396.28			
Regional States	Among Groups	3	80666.18	16.25Va	1.18	FCT=0.012	Va and FCT (*P = 0.341*)
	Among populations within Groups	43	1035086.58	1059.80Vb	76.66	FSC=0.77	Vb and FSC (*P < 0.001*)
	Within populations	1009	309257.11	306.50 Vc	22.17	FST=0.78	Vc and FST (*P < 0.001*)
	Total	1055	1425009.88	1382.55			
Geographical origin	Among Groups	4	116601.45	21.24 Va	1.54	FCT=0.02	Va and FIS (*P = 0.312*)
	Among populations within Groups	42	987329.87	1041.55 Vb	75.42	FSC=0.77	Vb and FSC (*P < 0.001*)
	Within populations	1009	321078.55	318.21 Vc	23.04	FST=0.76	Vc and FST(*P < 0.001*)
	Total	1055	1425009.88	1381.01			
Bayesian Model-based (STRUCTURE)	Among populations	4	531568.39	684.75	44.50	*FST=0.45*	*Va and FST (P < 0.001)*
	Among individuals within populations	523	870045.48	809.63	52.62	*FIS=0.95*	*Vb and FIS(P < 0.001)*
	Within individuals	528	23396	44.31	2.88	FIT=0.97	Vc and FIT (*P < 0.001*)
	Total	1055	1425009.88	1416.39			

DF†, Degree of freedom; SS, Sum of Squares; VC, Variance components; PV, Percentage of Variance; FI, Fixation index; ST, Significance test (at 1023 Permutations).

The *F_st_*-based pairwise genetic differentiation analysis for all pairs of populations revealed *F_st_* values ranging from 0 to 1, with a mean *F_st_* value of 0.76. There was significant differentiation between all pairs of populations, except in the case of NSH1 vs. BL2, WSH2 vs. WSH7, WSH2 vs. ESH2, MIRSH2 vs. NO, NO vs. NSH7, NSH6 vs. AR3 and AR3 vs. SW2 (**Figure 5B**; **Supplementary Table 6**). The historical rates of gene flow (Nm) for pairs of populations varied from 0 to 534.2, with a mean value of 0.85 (**Supplementary Table 7**). Of the populations considered in this study, WG, AR4, NSH8, SW3, NSH1, and WSH1 were the most distinct (**Figure 5B**; **Supplementary Table 6**). In contrast, NSH5, NG3, and WSH7 were the least differentiated populations across all pairs (*F_st_* = 0.47). Wide variation and significant Nei’s mean number of pairwise differences between populations (π_xy_) were revealed for all population pairs, except for NSH1 vs. BL2, WH vs. NO, NSH7 vs. AR3 and WH vs. NSH7 (**Figure 5C**, Green above diagonal, **Supplementary Table 8**). The Nei’s mean number of pairwise differences (π) within the populations varied from 0 (WH2) to 1861.99 (modern cultivars), thereby suggesting large differences between the populations according to their within-population genetic variation (**Figure 5B**, diagonal, **Supplementary Table 8**).

## 4 Discussion

### 4.1 Levels of SNP polymorphism

Durum wheat landraces have been grown for thousands of years and have been subjected to natural and human selection, resulting in their adaptation to various environmental conditions (Mengistu et al., 2016; Baloch et al., 2017). Locally adapted germplasm, however, have been lost sporadically due to their replacement by new cultivars developed through modern breeding for specific traits (Mengistu et al., 2016; Pont et al., 2019; Mazzucotelli et al., 2020; Sansaloni et al., 2020; Sthapit et al., 2020). Hence, this scenario demands revisiting the crop’s wild relatives and landraces, which are the primary genetic sources for transferring valuable alleles required to boost genetic variation in the cultivars, to cope with unpredictable challenges arising from changing climates (Kabbaj et al., 2017; Kilian et al., 2020; Adhikari et al., 2022). This study has provided a more profound insight into the population structure and genetic relationships in durum wheat gene pools collected from different eco-geographic regions of Ethiopia.

The physical distribution of selected SNPs was revealed in this study, with the highest number of SNPs present in the B genome than in the A genome. Previous research also revealed more SNPs on the B genome than on the A genome in the genetic diversity study of durum wheat (Alipour et al., 2017; Baloch et al., 2017; Kabbaj et al., 2017; Rufo et al., 2019; Alemu et al., 2020; Negisho et al., 2021). However, gene diversity and PIC indices were not significantly different between the A and B genomes regardless of the fact that Ethiopian durum wheat collections showed a high level of genetic variation. The result suggests that the average mutation rates of the A and B genomes in Ethiopian durum wheat landraces are comparable. The data support previous research findings on Ethiopian durum wheat landraces and cultivars (Mengistu et al., 2016; Alemu et al., 2020).

Compared to some previous research, the present study showed high mean gene diversity (0.34) and PIC (0.41), indicating the high genetic variation in Ethiopian durum wheat, which might have arisen due to crucial evolutionary forces such as mutation rate, natural selection, linked selection, population history, and demographic history. Previous research (Harlan,1969; Pecetti et al.,1992; Mengistu et al., 2015; Kabbaj et al., 2017) reported the uniqueness and high genetic diversity in Ethiopian durum wheat landraces compared to germplasm sources from different sites, which could be attributed due to the long-term separation of Ethiopian durum wheat landraces from primary sources of origin and internal germplasms sources. For instance, Alemu et al. (2020) reported mean gene diversity and PIC of 0.25 and 0.20, respectively, using 192 Ethiopian durum wheat landraces consisting of 167 landraces and 25 modern cultivars genotyped with 15,338 SNP markers. Likewise, Ren et al. (2013) reported mean gene diversity and PIC of 0.22 and 0.18 using 150 worldwide durum wheat landraces genotyped with 1,536 SNP markers. In other research on durum wheat germplasm diversity, lower magnitudes of gene diversity and PIC were noted compared to those obtained in the present study (Baloch et al., 2017; Kabbaj et al., 2017; Rufo et al., 2019; Mahboubi et al., 2020; Mazzucotelli et al., 2020).

The Ethiopian durum wheat gene pool exhibits high mean gene diversity and PIC values at the A subgenome, B subgenome, and whole genome levels. These results are in line with previous research that showed high genetic diversity in Ethiopian durum wheat germplasm (Mengistu et al., 2018; Alemu et al., 2020; Negisho et al., 2021). There is also a widely accepted understanding by several scholars that broad adaptation of germplasm to different agroecology, diverse farmers’ agricultural practices, and natural cross-pollination facilitated by farmers’ practices of planting mixed genotypes could have resulted in high genetic diversity (Peterson et al., 2014; Mengistu et al., 2015; Alemu et al., 2020).

### 4.2 Magnitude and pattern of within populations allelic diversity

Genetic diversity parameters mean of GD (0.10), I (0.11), %P (30.00%), and He (0.07) of the loci recorded low variation within the durum wheat landraces and is by far below those reported previously (Mengistu et al., 2016; Alemu et al., 2020; Negisho et al., 2021). The differences could be attributed to differences in sample size as well as differences in genetic background between the landraces used in this study and those used in previous studies. The low diversity within accessions of most of the landraces is primarily due to the fact their alleles were fixed across most of the loci. Hence, a single genotype could potentially provide sufficient genetic information in such accessions. However, some landraces (15 of those included in this study) showed high genetic variation within the accessions. Since genetic information generated based on a single plant of such landraces cannot sufficiently explain their genetic makeup, each of them should be represented by multiple individuals in genomic research to draw acceptable conclusions. The low estimate of mean gene flow (0.08) and broad variation in fixation indices (*F_IS_, F_IT_*, and *F_st_*) suggest a high degree of genetic differentiation among the landraces and limited gene exchange, as reported previously (Rufo et al., 2019; Mourad et al., 2020; Negisho et al., 2021). Low within-landrace genetic variation and wide variation in fixation indices were also reported in sorghum landraces from Ethiopia (Enyew et al., 2022).

A Hardy Weinberg Equilibrium (HWE) test is a widely used approach to estimate allelic and genotype frequencies in populations, thereby providing crucial information regarding reproductive mechanisms as well as the different evolutionary forces shaping their genetic makeups. The HWE test for individual landraces revealed that the vast majority of the loci are not in HWE. This is not surprising, as durum wheat reproduces primarily through self-fertilization (Hucl and Matus-Cádiz, 2001). Several evolutionary factors could also influence this result, including gene flow, natural and artificial selection, mutation, population size, and different degrees of outcrossing. However, for some landrace populations, including NSH6, WGM2, NO, NSH2, AR1, and BL1, more than half of the polymorphic loci did not significantly deviate from HWE. These indicate the need for further research to gain deeper insight into the diversity in the reproductive mechanisms of durum wheat. Several research findings indicate that the outcrossing rates of durum wheat range from 0 to 6.7% (Hucl and Matus-Cádiz, 2001).

### 4.3 Pattern and extent of linkage disequilibrium (LD)

Determining the extent, pattern, and distribution of LD throughout the durum wheat genome provides crucial information necessary to define inherited genomic regions (Sajjad et al., 2012; Roncallo et al., 2021). Furthermore, the extent and pattern of LD in germplasm guide the mapping resolution of targeted genomic regions and the strategies to decide whether to use coarse mapping based on a set of less diverse germplasm with lower SNP markers or fine mapping with a higher number of markers based on a set of genetically diverse germplasm (Gaut and Long, 2003; Sajjad et al., 2012). LD has been estimated using several types of DNA markers in durum wheat (Maccaferri et al., 2005; Laidò et al., 2014; Taranto et al., 2020). This study revealed 30.39% (*r^2^* ≥ 0.2*, p*<0.01) significant SNP pairs across the durum wheat genome, a considerably higher percentage in comparison with the 13.4% (*p*<0.01) reported by Roncallo et al. (2021), 27.6% (*p*<0.01) by Mekonnen et al. (2021), and 19.8% (*p*<0.01) by Mulugeta et al. (2023).

Compared to previous research (Alemu et al., 2020; Mekonnen et al., 2021), a high genomic mean *r^2^ = *0.21 (all linked SNP pairs in LD, *p*<0.01) was estimated for the entire durum wheat set used in this study, including both landraces and cultivars. These results demonstrate the influence of significant elements of LD because of genetic linkage and the residual LD that might arise due to factors such as selection, rate of genetic recombination, and evolutionary history, leading to high genetic diversity (Fayaz et al., 2019; Roncallo et al., 2021). In agreement with previous research on the pattern and extent of LD in durum wheat (Maccaferri et al., 2019; Alemu et al., 2020; Taranto et al., 2020; Roncallo et al., 2021), this study also revealed distinct variation in the pattern and LD decay distances across each of the chromosomes and genomic regions of durum wheat.

The LD decay (at cut-off *r^2^ = *0.2) declined within the physical distance varying from 3.65 Mbp (chromosome 4A) to 22.90 Mbp(chromosome 3B), with a mean of 8.56 Mbp across the genome is comparable with previous research, i.e., 11.8 Mbp by Roncallo et al. (2021), 9.6 Mbp by Wang et al. (2019), and 9.96 Mbp by Taranto et al. (2020). However, this result is far below the previous report by Alemu et al. (2020) using Ethiopian durum wheat landraces (69.1 Mbp) and Bassi et al. (2019) using three different sets of durum wheat germplasm (51.3 Mbp). The differences could arise from the type and density of markers covering genomic regions and evolutionary forces acting on the germplasm.

### 4.4 Pattern of nucleotide variation across the genome

The high nucleotide diversity (π) and Tajima’s D revealed in this study suggest substantial genetic variation in Ethiopian durum wheat populations. The mean π and Tajima’s D values across the whole genome of 0.33 and 4.43, respectively, are high compared to several previous reports (Akhunov et al., 2010; Cavanagh et al., 2013; Liu et al., 2019). Reduced levels of genetic diversity were observed in the pericentromeric regions of most of the chromosomes except in chromosomes 1A, 1B, 6A, and 6B. These are similar to the reports of a genome-wide diversity scan of durum germplasm by Akhunov et al. (2010); Maccaferri et al. (2019), and Liu et al. (2019). However, chromosomes 1A, 1B, 6A, and 6B showed widespread variation across genomic regions suggesting that the influence of intense selection and domestication pressures on these chromosomes is minimal. The distal regions of all chromosomes showed higher genomic variation than the proximal regions and indicated the occurrence of balancing selections in these regions, in agreement with previous research in wheat (Zhou et al., 2018; Liu et al., 2019; Maccaferri et al., 2019; Gaire et al., 2020; Mazzucotelli et al., 2020). Zhou et al. (2018) indicated that near or in the centromeric regions, there is nearly 0 gene content and meiotic recombination in cereals’ chromosomes, thus resulting in low genetic variation in the regions.

### 4.5 Selection signatures and associated putative genes

Previous research indicated that the selection scan approach based on the genetic differentiation (*Fst_)_* outlier test is suitable to identify genomic regions subjected to selection signatures because it is not strongly influenced by ascertainment bias (Foll and Gaggiotti, 2008; Cavanagh et al., 2013). The *F_st_* outlier test identified 85 selection signatures that spread across all chromosomes. However, the number of selection signatures identified in this study is far below the signals revealed in previous investigations in wheat, thereby indicating that the influence of selection during or after domestication by farmers and breeding on Ethiopian durum wheat landraces is low when compared to germplasm from other parts of the world. For instance, Liu et al. (2019), using 687 Chinese and Pakistan landraces and cultivars genotyped with a 90K SNP array, found 268, 318, and 109 genomic regions in germplasm from China, Pakistan, and both, respectively. Zhou et al. (2018) also identified 148 loci associated with grain yield and host plant tolerance to pathogens using 717 Chinese wheat landraces genotyped with 27,933 DArT and 312,831 SNP markers. Additionally, Cavanagh et al. (2013) observed 308 loci associated with yield potential, vernalization, and plant height based on 2,994 wheat germplasm genotyped with 6,305 SNPs.

Consistent with previous research (Zhou et al., 2018; Liu et al., 2019), more selection signatures were identified on the B genome than on the A genome in this study. This indicates that the B genome carries more adaptation, agronomic, and domestication trait-related genes than the A genome. Likewise, this shows that the selection pressure that influenced the B genome during or after domestication by farmers and breeders was stronger than its influence on the A genome. The putative candidate genes identified near or within the regions under selection were associated with several desirable traits in wheat. Several known quantitative trait loci (QTL) for grain yield (Roncallo et al., 2018), plant height (Roncallo et al., 2017), leaf rust resistance (Aoun et al., 2016), yellow rust resistance (Liu et al., 2017), stem rust resistance (Letta et al., 2014), primary root length and heading date (Maccaferri et al., 2008; Maccaferri et al., 2016; Giunta et al., 2018), grain protein content (Suprayogi et al., 2009), test weight (Canè et al., 2014), grain β-glucan content (Marcotuli et al., 2017), and phenolic acid contents (Nigro et al., 2017) were found to be co-localized and associated with the genomic regions influenced by selection signatures as revealed in this study.

### 4.6 Genetic population structure and relationship

A fundamental component of harnessing genetic diversity is understanding the genetic population structure, which provides crucial information regarding available genetic resources, thereby contributing to the development of future conservation strategies and broadening the genetic base of crops (Eltaher et al., 2018; Tehseen et al., 2022). The model-based clustering using STRUCTURE revealed the highest delta K (ΔK) at K = 2, followed by K = 5, thereby suggesting a possible number of subpopulations. As previously reported, if a value of K = 2 is found in STRUCTURE analyses, it may indicate the inability of the STRUCTURE algorithm to estimate the population structure appropriately (Janes et al., 2017; Tehseen et al., 2022). Hence, we chose K = 5 as an optimal number of subpopulations representing the 528 genotypes, which showed up to 80% concordance with the PCoA and UPGMA-based analyses.

The grouping of the diverse landraces into five distinct clusters using the PCoA, UPGMA, and STRUCTURE suggests that they had evolved from different gene pools or they are the results of independent events shaped by different evolutionary forces (genetic drift, mutation, migration, selection, and in flux/out flux of genes in the form of germplasm exchange) that separated them into different gene pools. UPGMA tree cluster 1 (Cl-I) comprised 25 landraces grouped together with all modern cultivars. This could have be caused by the fact that some farmers practice planting mixed genotypes, allowing cross-pollination between cultivars and landraces. Another probable reason could be that cultivars were be mistakenly classified as landraces during the germplasm collecting mission or that they are admixed germplasm. Negisho et al. (2021) obtained similar results using 285 durum wheat landraces. The admixture level in this cluster was high, thus indicating that almost all breeding programs in Ethiopia utilized germplasm obtained from the Centro Internacional de Mejoramiento de Maíz y Trigo (CIMMYT, Mexico) and the International Center for Agricultural Research in the Dry Areas (ICARDA, Syria) as a source of desirable genotypes in the variety development pipeline to broaden the genetic basis of national breeding programs.

### 4.7 Genetic differentiation of the hierarchical populations

AMOVA indicated significant genetic differences among landraces, showing that genetic variation between populations is more significant than genetic variation within populations. Observed genetic variation among individuals within landraces might have occurred during domestications or might have been caused by seed exchange among farmers and local traders from adjoining and nonadjacent regions. Alemu et al. (2020) found higher genetic variation between the two groups (61.02%) than among individuals within the group (38.98%) using 167 landraces and 25 cultivars from Ethiopia. Similarly, Kabbaj et al. (2017) and Roncallo et al. (2021) reported higher genetic variation between sub-populations than among individuals within subpopulations using different durum wheat populations.

### 4.8 The implication of this study for durum wheat breeding

Genetic characterization of the diverse set of durum wheat germplasm provided a sound insight into the population structure and genetic diversity of Ethiopian durum wheat gene pool as well as the genetic linkages between the SNP markers along its chromosomes. The information provided here facilitates the identification of beneficial loci and useful alleles that will aid in the development of more resilient durum wheat cultivars capable of coping with climate change challenges and ensuring durum wheat’s significant role in sustainable food security. These accumulated beneficial genetic variants of Ethiopian durum wheat could also help breeders to exploit available genetic variation more efficiently, optimizing future yield potential in more sustainable production systems and driving further discovery and deployment of beneficial alleles. The genetic analyses based on LD, GD, ND, Tajima’s D, and loci under selection revealed key genomic information, including apparent differences among the landraces. This provides a basis for future conservation of the crop’s genetic resources and breeding efforts to improve the crop.

## 5 Conclusion

The Illumina Infinium 25k wheat SNP array was used for genotyping 528 Ethiopian durum wheat to assess genetic diversity and population structure, determine LD, and uncover selection signatures related to domestication and breeding. High nucleotide diversity and Tajima’s D were observed at distal regions than pericentromeric regions (nearly zero diversity) of the chromosomes except for 1A, 1B, 6A, and 6B, which showed high diversity across their entire regions indicating the influence of selection during domestication by farmers and breeders for specific traits. Loci found under balancing selection spanned over all 14 durum chromosomes, whereas those under directional selection were distributed across 2A, 3A, 5B, 6B, and 7B chromosomes. Interestingly, genomic regions previously reported to impact grain yield, days to heading, grain quality, and disease resistance have been confirmed in this study. Hence, our results showed Ethiopian durum wheat germplasm’s high genetic diversity and untapped potential, which can be explored to discover novel genes for broadening the gene pool to develop climate-resilient cultivars. We recommend that Durum wheat breeders should strive to use these genetic materials to develop improved cultivars through fine mapping of genetically complex traits like grain yield and end-use quality traits, thereby maintaining yield stability, genetic gain, and adaptation to specific biotic and abiotic factors.

## Data availability statement

The original contributions presented in the study are included in the article/**Supplementary Material**. Further inquiries can be directed to the corresponding author.

## Author contributions

Conceptualization: BM, MG, KT, RO, and TH, Methodology: BM, MG, KT, and RO, Data curation: BM, Formal analysis: BM, Visualization: BM, Investigation: BM and MG, Resources: KT, RO, MG, and TH, Funding acquisition: KT, RO, MG, and TH, Project administration: KT, RO, MG, TH, and CH, Supervision: KT, RO, MG, TH, CH, and FH, Writing original draft: BM, Writing-review, and editing: BM, RO, MG, KT, TH, CH, and FH. All authors contributed to the article and approved the submitted version.

## Supplementary material

Supplementary Figure 1
**(A).** The pattern of site frequency spectrum based on the proportion of the minor allele frequency (MAF) of single nucleotide polymorphism (SNP) in the populations EH1, NSH1, WSH1, WSH3, WH, WGM1, WGM2, WSH2, AR1, BL1, NSH2, NG1, NG2, ESH1 and WSH4 of the 47 durum wheat landraces. **(B).** The pattern of site frequency spectrum based on the proportion of the minor allele frequency (MAF) of single nucleotide polymorphism (SNP) in the populations NW, BL2, NO, JM, NG3, ESH2, NSH3, SM, WSH5, WSH6, WSH7, NSH4, ESH3, NSH5 and NSH6 of the 47-durum wheat landraces. **(C).** The pattern of site frequency spectrum based on the proportion of the minor allele frequency (MAF) of single nucleotide polymorphism (SNP) in the populations TG1, NSH7, NSH8, EH3, AR2, AR3, NSH9, SW1, SW2, EGM, AR4, WSH8, WSH9, SW3 and MC of the 47 durum wheat landraces.

## References

[B1] Adhikari S. Kumari J. Jacob S. R. Prasad P. Gangwar O. P. Lata C.. (2022). Landraces-potential treasure for sustainable wheat improvement. Genet. Resour. Crop Evol. 69, 499–523. doi: 10.1007/s10722-021-01310-5

[B2] Akhunov E. D. Akhunova A. R. Anderson O. D. Anderson J. A. Blake N. Clegg M. T.. (2010). Nucleotide diversity maps reveal variation in diversity among wheat genomes and chromosomes. BMC Genomics 11 (1), 1–22. doi: 10.1186/1471-2164-11-702 21156062 PMC3022916

[B501] Alaux M. Rogers J. Letellier T. Flores R. Pommier C. Mohellibi N.. (2018). “The IWGSC Data repository and wheat data resources hosted at URGI: Overview and perspectives,*Triticum turgidum* ssp. durum)” in Proceedings of the PAG XXVI-Plant and Animal Genome Conference, San Diago, CA. 7

[B3] Alemu A. Feyissa T. Letta T. Abeyo B. (2020). Genetic diversity and population structure analysis based on the high density SNP markers in Ethiopian durum wheat (*Triticum turgidum* ssp. durum). BMC Genet. 21 (1), 1–12. doi: 10.1186/s12863-020-0825-x 32050895 PMC7017545

[B4] Alipour H. Bihamta M. R. Mohammadi V. Peyghambari S. A. Bai G. Zhang G. (2017). Genotyping-by-sequencing (GBS) revealed molecular genetic diversity of Iranian wheat landraces and cultivars. Front. Plant Sci. 8. doi: 10.3389/fpls.2017.01293 PMC5583605 28912785

[B5] Aoun M. Breiland M. Kathryn T. M. Loladze A. Chao S. Xu S. S.. (2016). Genome-wide association mapping of leaf rust response in a durum wheat worldwide germplasm collection. Plant Genome 9 (3). doi: 10.3835/plantgenome2016.01.0008 27902791

[B6] Asmamaw M. Keneni G. Tesfaye K. (2019). Genetic diversity of ethiopian durum wheat (Triticum durum desf.) landrace collections as reveled by SSR markers. Adv. Crop Sci. Technol. 7 (1), 413. doi: 10.4172/2329-8863.1000413

[B500] Badaeva E. D. Konovalov F. A. Knüpffer H. Fricano A. Ruban A. S. Kehel Z.. (2019). Genetic diversity, distribution and domestication history of the neglected GGAtAt genepool of wheat. Theor. Appl. Genet. 135 (3), 755–776. doi: 10.1007/s00122-021-03912-0 PMC8942905 34283259

[B8] Baloch F. S. Alsaleh A. Shahid M. Q. Çiftçi V. Sáenz De Miera L. E. Aasim M.. (2017). A whole genome DArTseq and SNP analysis for genetic diversity assessment in durum wheat from central fertile crescent. PLOS One 12 (1), e0167821. doi: 10.1371/journal.pone.0167821 28099442 PMC5242537

[B9] Bassi F. M. Brahmi H. Sabraoui A. Amri A. Nsarellah N. Nachit M. M.. (2019). Genetic identification of loci for Hessian fly resistance in durum wheat. Mol. Breed. 39, 1–16. doi: 10.1007/s11032-019-0927-1

[B11] Bradbury P. J. Zhang Z. Kroon D. E. Casstevens T. M. Ramdoss Y. Buckler E. S. (2007). TASSEL: Software for association mapping of complex traits in diverse samples. Bioinformatics 23, 2633–2635. doi: 10.1093/bioinformatics/btm308 17586829

[B1000] Breseghello F. Sorrells M. E. (2006). Association analysis as a strategy for improvement of quantitative traits in plants. Crop Sci. 46 (3), 1323–1330. doi: 10.2135/cropsci2005.09-0305

[B12] Canè M. A. Maccaferri M. Nazemi G. Salvi S. Francia R. Colalongo C.. (2014). Association mapping for root architectural traits in durum wheat seedlings as related to agronomic performance. Mol. Breed. 34, 1629–1645. doi: 10.1007/s11032-014-0177-1 25506257 PMC4257993

[B13] Carović-Stanko K. Liber Z. Vidak M. Barešić A. Grdiša M. Lazarević B.. (2017). Genetic diversity of croatian common bean landraces. Front. Plant Sci. 8. doi: 10.3389/fpls.2017.00604 PMC5397504 28473842

[B14] Cavanagh C. R. Chao S. Wang S. Huang B. E. Stephen S. Kiani S.. (2013). Genome-wide comparative diversity uncovers multiple targets of selection for improvement in hexaploid wheat landraces and cultivars. Proc. Natl. Acad. Sci. U S A 10 (20), 8057–8062. doi: 10.1073/pnas.1217133110 PMC3657823 23630259

[B15] Dejene K. M. Mario E. P. E. (2016). Revisiting the ignored Ethiopian durum wheat (*Triticum turgidum* var. durum) landraces for genetic diversity exploitation in future wheat breeding programs. J. Plant Breed. Crop Sci. 8 (4), 45–59. doi: 10.5897/jpbcs2015.0542

[B16] Devlin B. Risch N. (1995). A comparison of linkage disequilibrium measures for fine-scale mapping. Genomics 29 (2), 311–322. doi: 10.1006/geno.1995.9003 8666377

[B17] Earl D. A. von Holdt B. M. (2012). STRUCTURE HARVESTER: A website and program for visualizing STRUCTURE output and implementing the Evanno method. Conserv. Genet. Resour 4, 359–361. doi: 10.1007/s12686-011-9548-7

[B18] Eltaher S. Sallam A. Belamkar V. Emara H. A. Nower A. A. Salem K. F. M.. (2018). Genetic diversity and population structure of F3:6 Nebraska Winter wheat genotypes using genotyping-by-sequencing. Front. Genet. 9. doi: 10.3389/fgene.2018.00076 PMC5857551 29593779

[B19] Enyew M. Feyissa T. Carlsson A. S. Tesfaye K. Hammenhag C. Geleta M. (2022). Genetic diversity and population structure of sorghum [Sorghum bicolor (L.) moench] accessions as revealed by single nucleotide polymorphism markers. Front. Plant Sci. 12. doi: 10.3389/fpls.2021.799482 PMC8766336 35069657

[B20] Eticha F. Belay G. Bekele E. (2006). Species diversity in wheat landrace populations from two regions of Ethiopia. Genet. Resour. Crop Evol. 53, 387–393. doi: 10.1007/s10722-004-6095-z

[B21] Evanno G. Regnaut S. Goudet J. (2005). Detecting the number of clusters of individuals using the software STRUCTURE: A simulation study. Mol. Ecol. 14 (8), 2611–2620. doi: 10.1111/j.1365-294X.2005.02553.x 15969739

[B22] Excoffier L. Lischer H. E. L. (2010). Arlequin suite ver 3.5: A new series of programs to perform population genetics analyses under Linux and Windows. Mol. Ecol. Resour. 10 (3), 564–567. doi: 10.1111/j.1755-0998.2010.02847.x 21565059

[B23] Fayaz F. Aghaee S. M. Talebi R. Azadi A. (2019). Genetic Diversity and Molecular Characterization of Iranian Durum Wheat Landraces (*Triticum turgidum* durum (Desf.) Husn.) Using DArT Markers. Biochem. Genet. 57, 98–116. doi: 10.1007/s10528-018-9877-2 30051349

[B24] Fiedler J. D. Salsman E. Liu Y. Michalak de Jiménez M. Hegstad J. B. Chen B.. (2017). Genome-wide association and prediction of grain and semolina quality traits in durum wheat breeding populations. Plant Genome 10 (3). doi: 10.3835/plantgenome2017.05.0038 29293807

[B25] Flint-Garcia S. A. Thornsberry J. M. Edward IV, S. B. (2003). Structure of linkage disequilibrium in plants. Annu. Rev. Plant Biol. 54 (1), 357–374. doi: 10.1146/annurev.arplant.54.031902.134907 14502995

[B26] Foll M. Gaggiotti O. (2008). A genome-scan method to identify selected loci appropriate for both dominant and codominant markers: A Bayesian perspective. Genetics 180 (2), 977–993. doi: 10.1534/genetics.108.092221 18780740 PMC2567396

[B27] Gaire R. Ohm H. Brown-Guedira G. Mohammadi M. (2020). Identification of regions under selection and loci controlling agronomic traits in a soft red winter wheat population. Plant Genome 13 (2). doi: 10.1002/tpg2.20031 PMC12807307 33016613

[B28] Gaut B. S. Long A. D. (2003). The lowdown on linkage disequilibrium. Plant Cell. 15 (7), 1502–1506. doi: 10.1105/tpc.150730 12837942 PMC526043

[B29] Giraldo P. Royo C. González M. Carrillo J. M. Ruiz M. (2016). Genetic diversity and association mapping for agromorphological and grain quality traits of a structured collection of durum wheat landraces including subsp. durum, turgidum and diccocon. PLOS One 11 (11), 1–24. doi: 10.1371/journal.pone.0166577 PMC5113043 27846306

[B30] Giunta F. De Vita P. Mastrangelo A. M. Sanna G. Motzo R. (2018). Environmental and genetic variation for yield-related traits of durum wheat as affected by development. Front. Plant Sci. 9 (8). doi: 10.3389/fpls.2018.00008 PMC5778143 29403518

[B31] Harlan J. R. (1969). Ethiopia: A center of diversity. Econ. Bot. 23 (4), 309–314. doi: 10.1007/BF02860676

[B32] Huang X. Wei X. Sang T. Zhao Q. Feng Q. Zhao Y.. (2010). Genome-wide asociation studies of 14 agronomic traits in rice landraces. Nat. Genet. 42, 961–967. doi: 10.1038/ng.695 20972439

[B502] Hucl P. Matus-Cadiz M.. (2001). Isolation distances for minimizing out-crossing in spring wheat. Crop Science 41 (4), 1348–1351. doi: 10.2135/cropsci2001.4141348x

[B33] Janes J. K. Miller J. M. Dupuis J. R. Malenfant R. M. Gorrell J. C. Cullingham C. I.. (2017). The K = 2 conundrum. Mol. Ecol. 26 (14), 3594–3602. doi: 10.1111/mec.14187 28544181

[B34] Jin L. Lu Y. Xiao P. Sun M. Corke H. Bao J. (2010). Genetic diversity and population structure of a diverse set of rice germplasm for association mapping. Theor. Appl. Genet. 121, 475–487. doi: 10.1007/s00122-010-1324-7 20364375

[B35] Johansson E. Henriksson T. Prieto-Linde M. L. Andersson S. Ashraf R. Rahmatov M. (2020). Diverse wheat-alien introgression lines as a basis for durable resistance and quality characteristics in bread wheat. Front. Plant Sci. 11. doi: 10.3389/fpls.2020.01067 PMC7379150 32765555

[B36] Kabbaj H. Sall A. T. Al-Abdallat A. Geleta M. Amri A. Filali-Maltouf A.. (2017). Genetic diversity within a global panel of durum wheat (*Triticum durum*) landraces and modern germplasm reveals the history of alleles exchange. Front. Plant Sci. 8, 1277. doi: 10.3389/fpls.2017.01277 28769970 PMC5513985

[B37] Kadkol G. P. Sissons M. (2016). Durum wheat overview. Ref Modul Food Sci. 44 (5), 538–551. doi: 10.1016/B978-0-08-100596-5.00024-X

[B38] Kidane Y. G. Gesesse C. A. Hailemariam B. N. Desta E. A. Mengistu D. K. Fadda C.. (2019). A large nested association mapping population for breeding and quantitative trait locus mapping in Ethiopian durum wheat. Plant Biotechnol. J. 17 (7), 1380–1393. doi: 10.1111/pbi.13062 30575264 PMC6576139

[B39] Kilian B. Dempewolf H. Guarino L. Werner P. Coyne C. Warburton M. L. (2020). Crop Science Crop Science special issue: Adapting agriculture to climate change: A walk on the wild side. Crop Sci. 61 (1), 32–36. doi: 10.1002/csc2.20418

[B40] Kopelman N. M. Mayzel J. Jakobsson M. Rosenberg N. A. Mayrose I. (2015). Clumpak: A program for identifying clustering modes and packaging population structure inferences across K. Mol. Ecol. Resour. 15 (5), 1179–1191. doi: 10.1111/1755-0998.12387 25684545 PMC4534335

[B41] Kumar D. Chhokar V. Sheoran S. Singh R. Sharma P. Jaiswal S.. (2020). Characterization of genetic diversity and population structure in wheat using array based SNP markers. Mol. Biol. Rep. 47, 293–306. doi: 10.1007/s11033-019-05132-8 31630318

[B42] Kumar S. Stecher G. Li M. Knyaz C. Tamura K. (2018). MEGA X: Molecular evolutionary genetics analysis across computing platforms. Mol. Biol. Evol. 35, 1547–1549. doi: 10.1093/molbev/msy096 29722887 PMC5967553

[B43] Laidò G. Marone D. Russo M. A. Colecchia S. A. Mastrangelo A. M. De Vita P.. (2014). Linkage disequilibrium and genome-wide association mapping in tetraploid wheat (*Triticum turgidum* L.). PLOS One 9 (4), e95211. doi: 10.1371/journal.pone.0095211 24759998 PMC3997356

[B44] Letta T. Olivera P. Maccaferri M. Jin Y. Ammar K. Badebo A.. (2014). Association mapping reveals novel stem rust resistance loci in durum wheat at the seedling stage. Plant Genome 7 (1). doi: 10.3835/plantgenome2013.08.0026

[B48] Liu W. Maccaferri M. Rynearson S. Letta T. Zegeye H. Tuberosa R.. (2017). Novel sources of stripe rust resistance identified by genome-wide association mapping in ethiopian durum wheat (*Triticum turgidum* ssp. durum). Front. Plant Sci. 8 (774). doi: 10.3389/fpls.2017.00774 PMC5427679 28553306

[B47] Liu K. Muse S. V. (2005). PowerMaker: An integrated analysis environment for genetic maker analysis. Bioinformatics 21, 2128–2129. doi: 10.1093/bioinformatics/bti282 15705655

[B46] Liu J. Rasheed A. He Z. Imtiaz M. Arif A. Mahmood T.. (2019). Genome-wide variation patterns between landraces and cultivars uncover divergent selection during modern wheat breeding. Theor. Appl. Genet. 132, 2509–2523. doi: 10.1007/s00122-019-03367-4 31139853

[B49] Louwaars N. P. (2018). Plant breeding and diversity: A troubled relationship? Euphytica 214 (7), 114. doi: 10.1007/s10681-018-2192-5 30996394 PMC6434984

[B51] Maccaferri M. El-Feki W. Nazemi G. Salvi S. Canè M. A. Colalongo M. C.. (2016). Prioritizing quantitative trait loci for root system architecture in tetraploid wheat. J. Exp. Bot. 67 (4), 1161–1178. doi: 10.1093/jxb/erw039 26880749 PMC4753857

[B52] Maccaferri M. Harris N. S. Twardziok S. O. Pasam R. K. Gundlach H. Spannagl M.. (2019). Durum wheat genome highlights past domestication signatures and future improvement targets. Nat. Genet. 51, 885–895. doi: 10.1038/s41588-019-0381-3 30962619

[B53] Maccaferri M. Sanguineti M. C. Corneti S. Ortega J. L. A. Salem M. Bort J.. (2008). Quantitative trait loci for grain yield and adaptation of durum wheat (*Triticum durum* Desf.) across a wide range of water availability. Genetics 178 (1), 489–511. doi: 10.1534/genetics.107.077297 18202390 PMC2206097

[B54] Maccaferri M. Sanguineti M. C. Mantovani P. Demontis A. Massi A. Ammar K.. (2010). Association mapping of leaf rust response in durum wheat. Mol. Breed. 26, 189–228. doi: 10.1007/s11032-009-9353-0

[B55] Maccaferri M. Sanguineti M. C. Noli E. Tuberosa R. (2005). Population structure and long-range linkage disequilibrium in a durum wheat elite collection. Mol. Breed. 15, 271–290. doi: 10.1007/s11032-004-7012-z

[B56] Mahboubi M. Mehrabi R. Naji A. M. Talebi R. (2020). Whole-genome diversity, population structure and linkage disequilibrium analysis of globally diverse wheat genotypes using genotyping-by-sequencing DArTseq platform. 3 Biotech. 10 (2), 48. doi: 10.1007/s13205-019-2014-z PMC6960278 32002339

[B57] Marcotuli I. Gadaleta A. Mangini G. Signorile A. M. Zacheo S. A. Blanco A.. (2017). Development of a high-density SNP-based linkage map and detection of QTL for β-Glucans, Protein Content, Grain yield per spike and heading time in durum wheat. Int. J. Mol. Sci. 18 (6), 1329. doi: 10.3390/ijms18061329 28635630 PMC5486150

[B58] Mazzucotelli E. Sciara G. Mastrangelo A. M. Desiderio F. Xu S. S. Faris J.. (2020). The global durum wheat panel (GDP): an international platform to identify and exchange beneficial alleles. Front. Plant Sci. 11. doi: 10.3389/fpls.2020.569905 PMC7779600 33408724

[B59] Mekonnen T. Sneller C. H. Haileselassie T. Ziyomo C. Abeyo B. G. Goodwin S. B.. (2021). Genome-wide association study reveals novel genetic loci for quantitative resistance to septoria tritici blotch in wheat (Triticum aestivum L.). Front. Plant Sci. 12. doi: 10.3389/fpls.2021.671323 PMC8500178 34630445

[B62] Mengistu D. K. Kidane Y. G. Catellani M. Frascaroli E. Fadda C. Pè M. E.. (2016). High-density molecular characterization and association mapping in Ethiopian durum wheat landraces reveals high diversity and potential for wheat breeding. Plant Biotechnol. J. 14 (9), 1800–1812. doi: 10.1111/pbi.12538 26853077 PMC5067613

[B60] Mengistu D. K. Kidane Y. G. Fadda C. Pè M. E. (2018). Genetic diversity in Ethiopian Durum Wheat (*Triticum turgidum* var durum) inferred from phenotypic variations. Plant Genet. Resour. Characterisation Util 16, 39–49. doi: 10.1017/S1479262116000393

[B61] Mengistu D. K. Kiros A. Y. Pè M. E. (2015). Phenotypic diversity in Ethiopian durum wheat (*Triticum turgidum* var. durum) landraces. Crop J. 3 (3), 190–199. doi: 10.1016/j.cj.2015.04.003

[B63] Mérida-García R. Liu G. He S. Gonzalez-Dugo V. Dorado G. Gálvez S.. (2019). Genetic dissection of agronomic and quality traits based on association mapping and genomic selection approaches in durum wheat grown in Southern Spain. PLOS One 14 (2), e0211718. doi: 10.1371/journal.pone.0211718 30811415 PMC6392243

[B64] Mondal S. Rutkoski J. E. Velu G. Singh P. K. Crespo-Herrera L. A. Guzman C.. (2016). Harnessing diversity in wheat to enhance grain yield, climate resilience, disease and insect pest resistance and nutrition through conventional and modern breeding approaches. Front. Plant Sci. 7. doi: 10.3389/fpls.2016.00991 PMC4933717 27458472

[B65] Mourad A. M. I. Belamkar V. Baenziger P. S. (2020). Molecular genetic analysis of spring wheat core collection using genetic diversity, population structure, and linkage disequilibrium. BMC Genomics 21, 434. doi: 10.1186/s12864-020-06835-0 32586286 PMC7318758

[B66] Mulugeta B. Tesfaye K. Geleta M. Johansson E. Hailesilassie T. Hammenhag C.. (2022). Multivariate analyses of Ethiopian durum wheat revealed stable and high yielding genotypes. PLOS One 17 (8), e0273008. doi: 10.1371/journal.pone.0273008 35976886 PMC9385061

[B67] Mulugeta B. Tesfaye K. Ortiz R. Johansson E. Hailesilassie T. Hammenhag C.. (2023). Marker-trait association analyses revealed major novel QTLs for grain yield and related traits in durum wheat. Front. Plant Sci. 13, 1009244. doi: 10.3389/fpls.2022.1009244 36777537 PMC9909559

[B68] Negisho K. Shibru S. Pillen K. Ordon F. Wehner G. (2021). Genetic diversity of Ethiopian durum wheat landraces. PLOS One 16 (2), e0247016. doi: 10.1371/journal.pone.0247016 33596260 PMC7888639

[B69] Nei M. (1973). Analysis of gene diversity in subdivided populations. Proc. Natl. Acad. Sci. USA 70, 3321–3323. doi: 10.1073/pnas.70.12.3321 4519626 PMC427228

[B70] Nei M. (1987). Molecular Evolutionary Genetics. (New York Chichester, West Sussex: Columbia University Press). doi: 10.7312/nei-92038

[B71] Nigro D. Laddomada B. Mita G. Blanco E. Colasuonno P. Simeone R.. (2017). Genome-wide association mapping of phenolic acids in tetraploid wheats. J. Cereal Sci. 75, 25–34. doi: 10.1016/j.jcs.2017.01.022

[B72] Peakall R. Huff P. E. S. R. (1995). Evolutionary implications of allozyme and RAPD variation in-diplbid populations of dioecious buffalograss Buckloe dactyloides. Mol. Ecol. 4 (2), 135–148. doi: 10.1111/j.1365-294X.1995.tb00203.x

[B73] Peakall R. Smouse P. E. (2012). GenALEx 6.5: Genetic analysis in Excel. Population genetic software for teaching and research-an update. Bioinformatics 28. doi: 10.1093/bioinformatics/bts460 PMC3463245 22820204

[B504] Pecetti L. Annicchiarico P. Damania A. B. (1992). Biodiversity in a germplasm collection of durum wheat. Euphytica 60, 229–238. doi: 10.1007/BF00039403

[B74] Peterson G. W. Dong Y. Horbach C. Fu Y. B. (2014). Genotyping-by-sequencing for plant genetic diversity analysis: A lab guide for SNP genotyping. Diversity 6 (4), 665–680. doi: 10.3390/d6040665

[B75] Pfeifer B. Wittelsbürger U. Ramos-Onsins S. E. Lercher M. J. (2014). PopGenome: An efficient swiss army knife for population genomic analyses in R. Mol. Biol. Evol. 31, 1929–1936. doi: 10.1093/molbev/msu136 24739305 PMC4069620

[B76] Pont C. Leroy T. Seidel M. Tondelli A. Duchemin W. Armisen D.. (2019). Tracing the ancestry of modern bread wheats. Nat. Genet. 51 (5), 905–911. doi: 10.1038/s41588-019-0393-z 31043760

[B77] Pritchard J. K. Stephens P. Donnelly P. (2000). Inference of population structure using multilocus genotype data. Genetics 155 (2), 945–959. doi: 10.1093/genetics/155.2.945 10835412 PMC1461096

[B78] R Development Core team (2021). R: a language and environment for statistical computing. Version 4.0.5 (Vienna, Austria: R Foundation for Statistical Computing). Available at: https://www.R-project.org/.

[B79] Ren J. Sun D. Chen L. You F. M. Wang J. Peng Y.. (2013). Genetic diversity revealed by single nucleotide polymorphism markers in a worldwide germplasm collection of durum wheat. Int. J. Mol. Sci. 14 (4), 7061–7088. doi: 10.3390/ijms14047061 23538839 PMC3645677

[B80] Roncallo P. F. Akkiraju P. C. Cervigni G. L. Echenique V. C. (2017). QTL mapping and analysis of epistatic interactions for grain yield and yield-related traits in *Triticum turgidum* L. var. durum. Euphytica 213 (12), 277. doi: 10.1007/s10681-017-2058-2

[B81] Roncallo P. F. Beaufort V. Larsen A. O. Dreisigacker S. Echenique V. (2018). Genetic diversity and linkage disequilibrium using SNP (KASP) and AFLP markers in a worldwide durum wheat (*Triticum turgidum* L. Var durum) collection. PLOS One 14 (6), e0218562. doi: 10.1371/journal.pone.0218562 PMC6741835 31251752

[B82] Roncallo P. F. Larsen A. O. Achilli A. L. Pierre C. Gallo C. A. Dreisigacker S.. (2021). Linkage disequilibrium patterns, population structure and diversity analysis in a worldwide durum wheat collection including Argentinian genotypes. BMC Genomics 22, 1–17. doi: 10.1186/s12864-021-07519-z 33820546 PMC8022437

[B83] Rozas J. Ferrer-Mata A. Sanchez-DelBarrio J. C. Guirao-Rico S. Librado P. Ramos-Onsins S. E.. (2017). DnaSP 6: DNA sequence polymorphism analysis of large data sets. Mol. Biol. Evol. 34 (12), 3299–3302. doi: 10.1093/molbev/msx248 29029172

[B84] Rufo R. Alvaro F. Royo C. Soriano J. M. (2019). From landraces to improved cultivars: Assessment of genetic diversity and population structure of Mediterranean wheat using SNP markers. PLOS One 14 (7), e0219867. doi: 10.1371/journal.pone.0219867 31306459 PMC6629082

[B85] Sajjad M. Khan S. H. Kazi A. M. (2012). The low down on association mapping in hexaploid wheat (*Triticum aestivum* L.). J. Crop Sci. Biotechnol. 15, 147–158. doi: 10.1007/s12892-012-0021-2

[B86] Sall A. T. Chiari T. Legesse W. Seid-Ahmed K. Ortiz R. Van Ginkel M.. (2019). Durum wheat (*Triticum durum* Desf.): Origin, cultivation and potential expansion in sub-saharan Africa. Agronomy 9 (5), 263. doi: 10.3390/agronomy9050263

[B87] Sansaloni C. Franco J. Santos B. Percival-Alwyn L. Singh S. Petroli C.. (2020). Diversity analysis of 80,000 wheat accessions reveals consequences and opportunities of selection footprints. Nat. Commun. 11 (1), 1471. doi: 10.1038/s41467-020-18404-w 32917907 PMC7486412

[B88] Savage M. Vavilov N. I. Love D. (1994). Origin and geography of cultivated plants. Geogr. Rev. 84 (4), 492–494. doi: 10.2307/215338

[B89] Serrote C. M. L. Reiniger L. R. S. Silva K. B. Rabaiolli S. M. Dos S. Stefanel C. M. (2020). Determining the Polymorphism Information Content of a molecular marker. Gene 726, 144175. doi: 10.1016/j.gene.2019.144175 31726084

[B503] Simmonds S. W.. (1993). Origin and Geography of Cultivated Plants, by N. I. Vavilov. xxxi + 498 pp. (Cambridge: Cambridge University Press(1992)). J. Agric. Sci. 120 (3), 419–420. doi: 10.1017/s0021859600076632

[B91] Siol M. Jacquin F. Chabert-Martinello M. Smýkal P. Le Paslier M. C. Aubert G.. (2017). Patterns of genetic structure and linkage disequilibrium in a large collection of pea germplasm. G3 Genes Genomes Genet. 7 (8), 2461–2471. doi: 10.1534/g3.117.043471 PMC5555454 28611254

[B92] Soriano J. M. Villegas D. Aranzana M. J. García Del Moral L. F. Royo C. (2016). Genetic structure of modern durum wheat cultivars and mediterranean landraces matches with their agronomic performance. PLOS One 11 (8), e0160983. doi: 10.1371/journal.pone.0160983 27513751 PMC4981446

[B93] Soriano J. M. Villegas D. Sorrells M. E. Royo C. (2018). Durum wheat landraces from east and west regions of the mediterranean basin are genetically distinct for yield components and phenology. Front. Plant Sci. 9. doi: 10.3389/fpls.2018.00080 PMC5809869 29472936

[B94] Sthapit S. R. Marlowe K. Covarrubias D. C. Ruff T. M. Eagle J. D. McGinty E. M.. (2020). Genetic diversity in historical and modern wheat varieties of the U.S. Pacific Northwest. Crop Sci. 60 (6), 3175–3190. doi: 10.1002/csc2.20299

[B95] Suprayogi Y. Pozniak C. J. Clarke F. R. Clarke J. M. Knox R. E. Singh A. K. (2009). Identification and validation of quantitative trait loci for grain protein concentration in adapted Canadian durum wheat populations. Theor. Appl. Genet. 119, 437–448. doi: 10.1007/s00122-009-1050-1 19462147

[B96] Tajima F. (1989). Statistical method for testing the neutral mutation hypothesis by DNA polymorphism. Genetics 123 (3), 585–595. doi: 10.1093/genetics/123.3.585 2513255 PMC1203831

[B97] Taranto F. D’Agostino N. Rodriguez M. Pavan S. Minervini A. P. Pecchioni N.. (2020). Whole genome scan reveals molecular signatures of divergence and selection related to important traits in durum wheat germplasm. Front. Genet. 217. doi: 10.3389/fgene.2020.00217 PMC7187681 32373150

[B98] Tascioglu T. Metin O. K. Aydin Y. Sakiroglu M. Akan K. Uncuoglu A. A. (2016). Genetic diversity, population structure, and linkage disequilibrium in bread wheat (*Triticum aestivum* L.). Biochem. Genet. 54, 421–437. doi: 10.1007/s10528-016-9729-x 27048293

[B99] Tehseen M. M. Tonk F. A. Tosun M. Istipliler D. Amri A. Sansaloni C. P.. (2022). Exploring the genetic diversity and population structure of wheat landrace population conserved at ICARDA genebank. Front. Genet. 13. doi: 10.3389/fgene.2022.900572 PMC9240388 35783289

[B100] Wang S. Xu S. Chao S. Sun Q. Liu S. Xia G. (2019). A genome-wide association study of highly heritable agronomic traits in durum wheat. Front. Plant Sci. 10. doi: 10.3389/fpls.2019.00919 PMC6652809 31379901

[B101] Weir B. S. (1997). Genetic data analysis II. Biometrics. 53, 392. doi: 10.2307/2533134

[B102] Weir B. S. Cockerham C. C. (1984). Estimating F-statistics for the analysis of population structure. Evol. (N Y) 38, 1358–1370. doi: 10.1111/j.1558-5646.1984.tb05657.x 28563791

[B103] Yadav I. S. Singh N. Wu S. Raupp J. Wilson D. L. Rawat N.. (2022). Exploring genetic diversity of wild and related tetraploid wheat species *Triticum turgidum* and *Triticum timopheevii* . J. Adv. Res. 48, 47–60. doi: 10.1016/J.JARE.2022.08.020 36084813 PMC10248793

[B104] Zaïm M. El Hassouni K. Gamba F. Filali-Maltouf A. Belkadi B. Sourour A.. (2017). Wide crosses of durum wheat (Triticum durum Desf.) reveal good disease resistance, yield stability, and industrial quality across Mediterranean sites. F Crop Res. 214, 219–227. doi: 10.1016/j.fcr.2017.09.007

[B105] Zhou Y. Chen Z. Cheng M. Chen J. Zhu T. Wang R.. (2018). Uncovering the dispersion history, adaptive evolution and selection of wheat in China. Plant Biotechnol. J. 16 (1), 280–291. doi: 10.1111/pbi.12770 28635103 PMC5785339

