# Supplementary figures and images for: Harnessing genome-wide genetic diversity, population structure and linkage disequilibrium in Ethiopian durum wheat gene pool
Source: Front Plant Sci. 2023 Jul 20;14:1192356. doi: 10.3389/fpls.2023.1192356 (PMC10400094; doi:10.3389/fpls.2023.1192356)

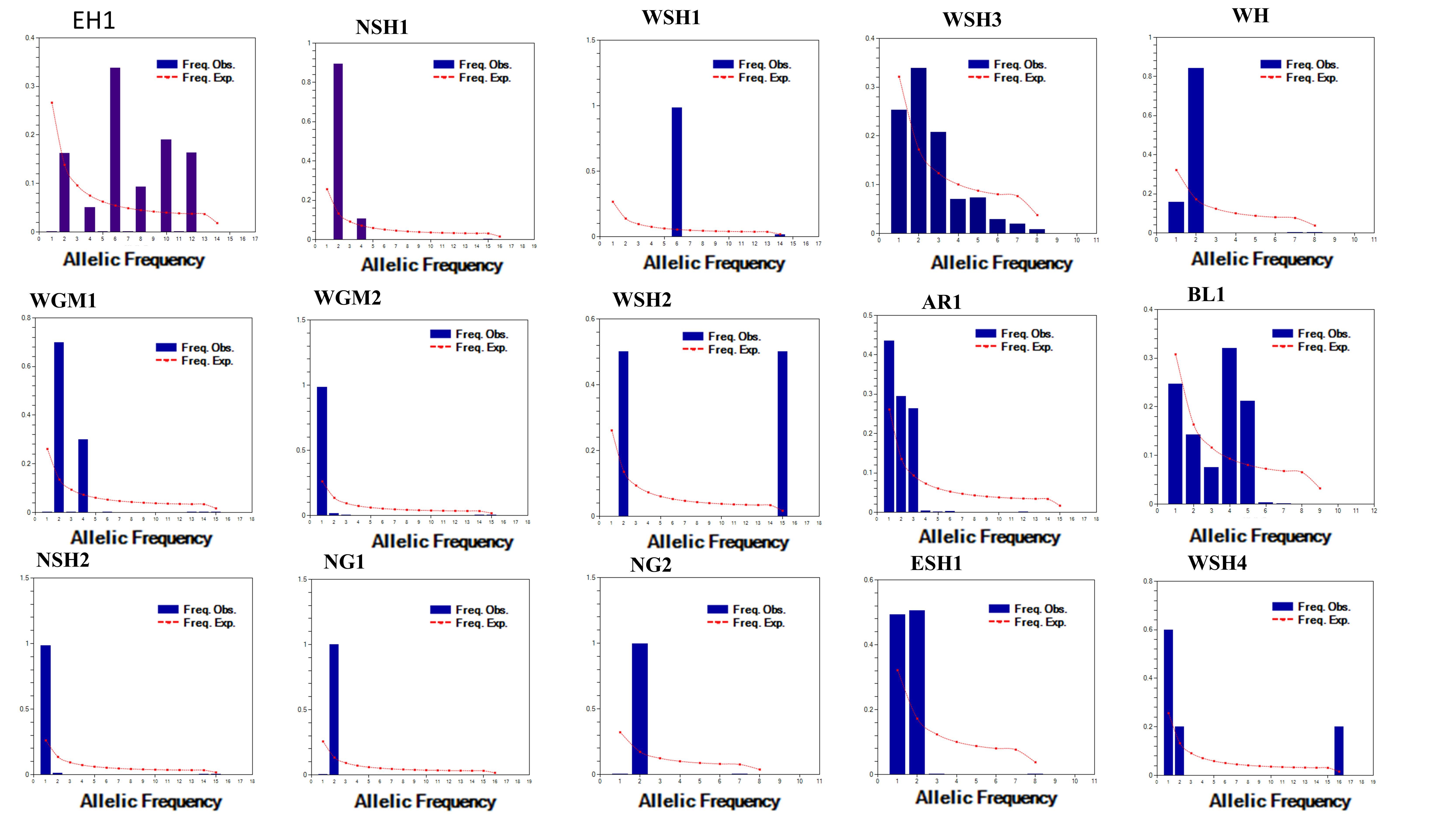

Supplement: Supplementary Figure 1 — (A). The pattern of site frequency spectrum based on the proportion of the minor allele frequency (MAF) of single nucleotide polymorphism (SNP) in the populations EH1, NSH1, WSH1, WSH3, WH, WGM1, WGM2, WSH2, AR1, BL1, NSH2, NG1, NG2, ESH1 and WSH4 of the 47 durum wheat landraces. (B). The pattern of site frequency spectrum based on the proportion of the minor allele frequency (MAF) of single nucleotide polymorphism (SNP) in the populations NW, BL2, NO, JM, NG3, ESH2, NSH3, SM, WSH5, WSH6, WSH7, NSH4, ESH3, NSH5 and NSH6 of the 47-durum wheat landraces. (C). The pattern of site frequency spectrum based on the proportion of the minor allele frequency (MAF) of single nucleotide polymorphism (SNP) in the populations TG1, NSH7, NSH8, EH3, AR2, AR3, NSH9, SW1, SW2, EGM, AR4, WSH8, WSH9, SW3 and MC of the 47 durum wheat landraces. [file Image_1.tif]

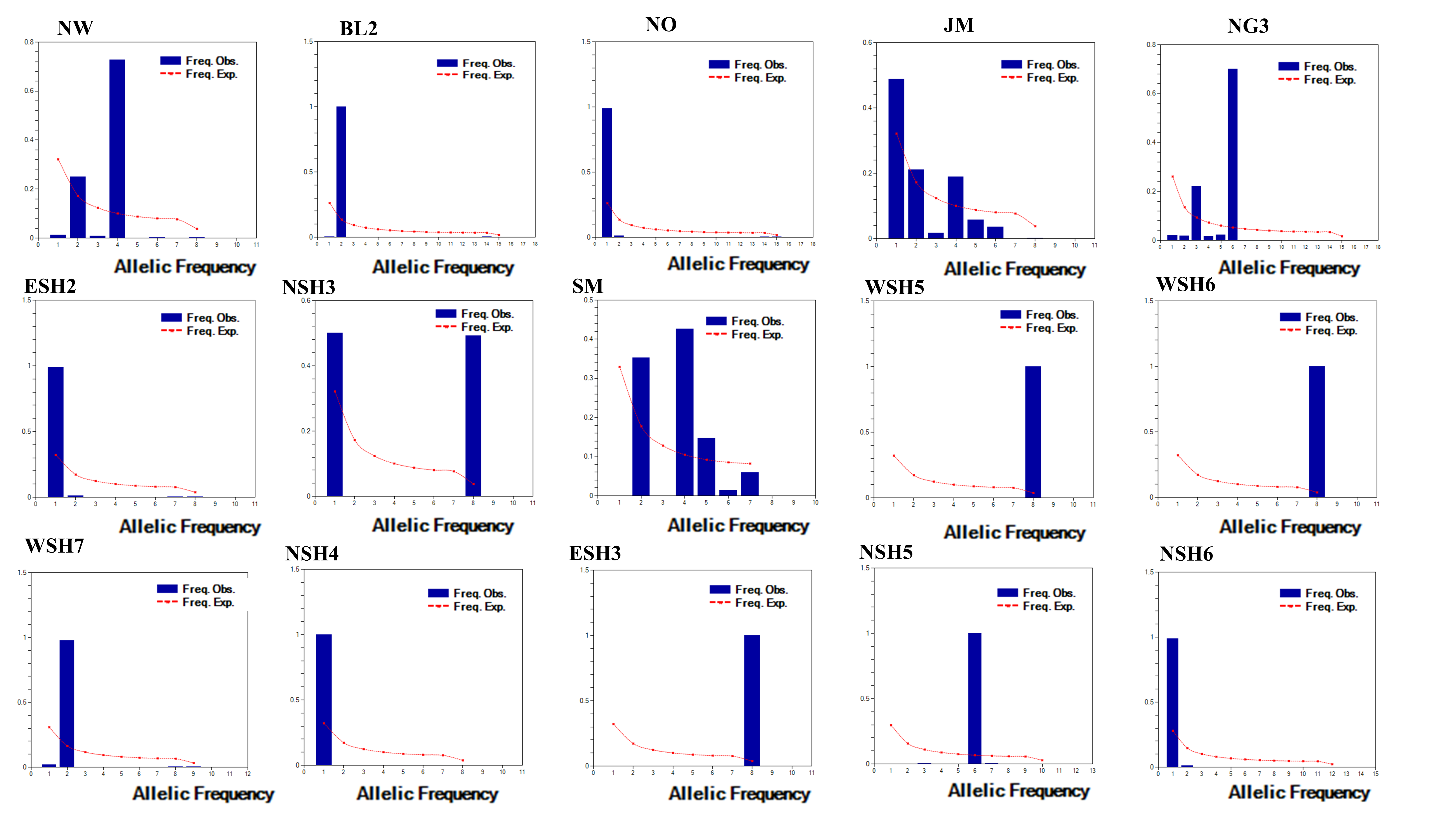

Supplement: Supplementary file 2 [file Image_2.tif]

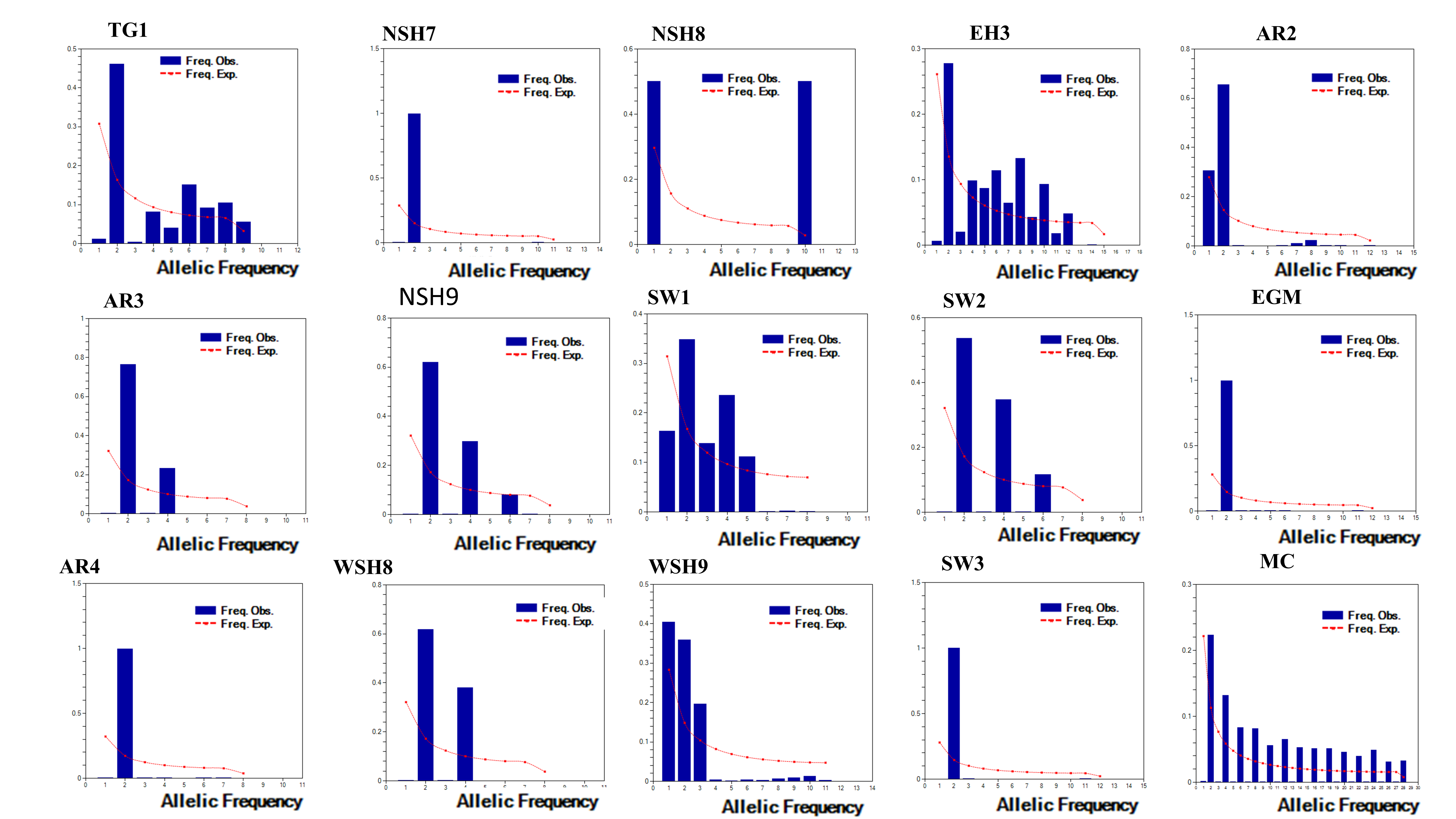

Supplement: Supplementary file 3 [file Image_3.tif]
